# Refrigerated amniotic membrane maintains its therapeutic qualities for 48 hours

**DOI:** 10.3389/fbioe.2024.1455397

**Published:** 2024-11-06

**Authors:** J. Stelling-Férez, J. M. Puente-Cuadrado, V. Álvarez-Yepes, M. Alcaraz, E. Tristante, I. Hernández-Mármol, I. Mompeán-Egea, A. M. García-Hernández, F. J. Nicolás

**Affiliations:** ^1^ Soluciones de Biología Molecular y Celular en Medicina Regenerativa, Health Sciences PhD Program, Universidad Católica de Murcia (UCAM), Murcia, Spain; ^2^ Regeneración Oncología Molecular y TGF-β, Instituto Murciano de Investigación Biosanitaria (IMIB)-Arrixaca, Hospital Clínico Universitario Virgen de la Arrixaca, Murcia, Spain; ^3^ Plataforma Sala Blanca Instituto Murciano de Investigación Biosanitaria (IMIB)-Pascual Parrilla, Murcia, Spain; ^4^ Hematopoietic Transplant and Cellular Therapy Unit, Virgen de la Arrixaca University Hospital, University of Murcia, Murcia, Spain

**Keywords:** amniotic membrane, conservation, clinical application, keratinocytes, chronification, cell migration, cell proliferation, wound healing

## Abstract

During wound healing, the migration of keratinocytes is critical for wound closure. The application of amniotic membrane (AM) on wounds with challenging contexts (e.g., chronification and diabetic foot ulcer) has proven very successful. However, the use of AM for clinical practice has several restraints when applied to patients; the most important restriction is preserving AM’s therapeutic properties between its thawing and application onto the patient’s wound. Moreover, AM collection and processing requires a cleanroom, together with specialized staff and equipment, and facilities that are not usually available in many hospitals and healthcare units. In this publication, we kept previously cryopreserved AM at different temperatures (37°C, 20°C, and 4°C) in different media (DMEM high glucose and saline solution with or without human albumin) and for long incubation time periods after thawing (24 h and 48 h). HaCaT keratinocytes and TGF-β1-chronified HaCaT keratinocytes were used to measure several parameters related to wound healing: migration, cell cycle arrest rescue, and the expression of key genes and migration-related proteins. Our findings indicate that AM kept in physiological saline solution at 4°C for 24 h or 48 h performed excellently in promoting HaCaT cell migration compared to AM that had been immediately thawed (0 h). Indeed, key proteins, extracellular signal-regulated kinase (ERK) and c-Jun, were induced by AM at 4°C in saline solution. Similarly, cell proliferation and different genes related to survival, inflammation, and senescence had, in all cases, the same response as to standard AM. These data suggest that the handling method in saline solution at 4°C does not interfere with AM’s therapeutic properties.

## Introduction

In human skin, wound healing is a fine-tuned process consisting of sequential cellular events that restore skin integrity (Ridiandries et al., 2018; Wang et al., 2018; Singer and Clark, 1999; Eming et al., 2014). This reparative process is crucial for skin homeostasis because this organ acts as a physical barrier against pathogens, ultraviolet radiation, and temperature (Nguyen and Soulika, 2019). Thus, complications in wound healing may cause an incomplete wound closure, leading to a continued inflammatory process, which produces a non-healing, chronic wound (Eming et al., 2007; Janis and Harrison, 2016). Chronic wounds cause the development of ulcers, lesions that severely compromise the patient’s quality of life, which get worse with diabetes or aging (Okonkwo and DiPietro, 2017; Blair et al., 2020; Bonifant and Holloway, 2019; Greenhalgh, 2003; Burgess et al., 2021; Patel et al., 2019). Indeed, the prevalence of ulcers in the elderly population is higher than the average in an increasingly aging society (Diaz-Herrera et al., 2021). As a consequence, complex wounds imply high costs and time-consuming treatments for healthcare systems (Kasuya and Tokura, 2014; Neuhaus et al., 2013; Ferreira et al., 2006; Pfeffer et al., 2004; Ball and Younggren, 2007).

Amniotic membrane (AM) is a well-studied perinatal derivative with multiple therapeutic effects on human-condition therapy, especially in tissue regeneration (Toda et al., 2007). Indeed, its current use in hospitals is considered an effective alternative and innovative therapy (Toda et al., 2007; Zelen et al., 2015; Flores et al., 2022; Gindraux et al., 2022). In previous publications, we have shown that AM enhances wound healing both *in vitro* and *in vivo* (Insausti et al., 2010; Alcaraz et al., 2015). Two critical cellular events for wound healing are keratinocyte migration from the wound edges and proliferation, two processes that AM remarkably potentiates (Singer and Clark, 1999; Alcaraz et al., 2015; Rodrigues et al., 2019). Thus, by using immortalized keratinocytes, HaCaTs, we have shown that AM promotes their migration from the wound edges of scratch assays and HaCaT rescue of a TGF-β1-arrested cell cycle (Alcaraz et al., 2015). Moreover, AM increases the motility of epithelial cells by enhancing the actin cytoskeleton and focal adhesion turnover (Bernabe-Garcia et al., 2017). The molecular mechanisms behind these AM-promoted effects are driven by epidermal growth factor (EGF) signaling, involving MAP kinase activation and the signaling contribution of TGF-β1 (Ruiz-Canada et al., 2018; Ruiz-Canada et al., 2021). A consequence of the influence of AM on these signaling pathways is the upregulation of the c-Jun transcription factor, a key regulator for cell migration (Herdegen et al., 1997; Li et al., 2003; Yue et al., 2020). Interestingly, we have shown that AM produces the reepithelization of complex wounds *in vivo*, evidenced by successful clinical trials where AM was used in patients with deep traumatic wounds and diabetic foot ulcers (DFUs) (Zelen et al., 2015; Insausti et al., 2010; Alcaraz et al., 2015; Castellanos et al., 2017; Pipino et al., 2022). Strikingly, we have observed that c-Jun transcription factor overexpression is induced by AM in those wounds to enhance the reepithelization of the skin (Insausti et al., 2010; Alcaraz et al., 2015). AM is particularly adequate for non-healing, chronic wounds, where the stimulation of different processes is produced in a context where cellular and molecular mechanisms are seriously affected due to the chronic, proinflammatory tissue context (Rodrigues et al., 2019; Ruiz-Canada et al., 2018; Ruiz-Canada et al., 2021). To reproduce this particular scenario in an *in vitro* system, we recently developed an *in vitro* model where HaCaT cells were serum-starved and simultaneously treated with TGF-β1 for 48 h (SSTC-HaCaT). These cells showed both an altered phenotype and a characteristic genetic profile, exhibiting cell cycle arrest and experiencing impaired cell migration and cell dynamics with an altered cell morphology (Liarte et al., 2020). Strikingly, AM treatment of SSTC-HaCaT cells was able to rescue their migration potential, improving cytoskeleton dynamics and regaining cell proliferation (Liarte et al., 2023). Therefore, these studies partly explain the therapeutic effects of AM on cells that are responsible for wound healing in different aspects.

Considering the above, AM features set this perinatal tissue as a promising treatment for wounds with complex non-healing contexts. However, it should be noted that AM extraction and processing are performed in a tissue bank with a cleanroom under proper safety and quality measures, conditions that are usually available only in central hospitals (Hofmann et al., 2023; Gramignoli et al., 2024). So far, the shipping and preparation of AM is still associated with great effort due to the deep-freeze storage and controlled thawing. Consequently, AM application in small healthcare centers may be limited, and the treatment of chronic wounds with AM becomes impractical. Indeed, many alternatives to the use of live-cryopreserved-stabilized AM have been developed, including frozen non-viable, desiccation, decellularization, conversion into a lyophilized powder, or freeze-drying (Koh et al., 2007; Thomasen et al., 2009; Thomasen et al., 2011; Xu et al., 2014; Jie et al., 2018; Davis et al., 2020; Svobodova et al., 2022). The method for preserving AM we favor the most, where AM is cryopreserved (−196°C), stabilized, and thawed, has serious technical and practical difficulties yet to be solved. To make the routine application of AM practicable for its use in chronic wounds, AM needs to be delivered in a way that respects and does not compromise its activity. The purpose of this research was to investigate *in vitro* therapeutic effects of AM subjected to different temperatures, incubation periods, and media storage conditions after thawing on HaCaT and SSTC-HaCaT cells.

## Methods

This study has been approved by the ethics committees at the University Clinical Hospital Virgen de la Arrixaca (Murcia, Spain) and the Spanish Agency for Drugs and Medical Devices (AEMPS). In every case, appropriate written informed consent was obtained from the AM donors.

### HaCaT cell culture and chronification

Immortalized HaCaT cells were grown in complete Dulbecco’s modified Eagle high-glucose medium (DMEM; Biowest, Nuaillé, France); this was DMEM supplemented with 10% fetal bovine serum (FBS; Gibco, Thermo Fisher Scientific, Rockford, IL, United States of America), 1% penicillin/streptomycin, and 1% L-glutamine (both from Biowest, Nuaillé, France). HaCaT cells were cultured in an incubator at 37°C with a 7.5% CO_2_ humidified atmosphere (Bernabe-Garcia et al., 2017; Ruiz-Canada et al., 2018; Liarte et al., 2020; Liarte et al., 2023). The HaCaT cell line was obtained from the London Institute of Cancer Research, UK, as a gift from Caroline S. Hill.

Chronification of HaCaT cells was performed as previously described in Liarte et al. (2020) and Liarte et al. (2023). HaCaT cells were grown until reaching 50% confluence, and the medium was replaced by FBS-free DMEM. At this point, cells were stimulated with 2 ng/mL of TGF-β1 (Peprotech, Rocky Hill, NJ, United States of America), and, in parallel, other cells were maintained in the FBS-free media for 48 h. In the case of cells stimulated with TGF-β1, this growth factor was added again at 24 h and 48 h. After 48 h, TGF-β1-treated HaCaT cells were designed as SSTC-HaCaT (serum-starved TGF-β1 chronified HaCaT), while untreated cells were designed as SS-HaCaT (serum-starved HaCaT).

### Amniotic membrane processing and treatments

The amniotic membrane was mechanically peeled from the chorion under sterile conditions, placed in a container with 1,000 mL of physiological saline solution (PSS) supplemented with cotrimazole (48 mg), tobramycin (50 mg), and vancomycin (50 mg), and rapidly transferred to the laboratory at room temperature. Under a laminar flow cabinet, the amnion was washed four times with 100 mL of 0.9% saline solution. After washing and before cryopreservation, the AM was placed on a sterile cloth and cut into fragments of approximately ≤8.5 cm × 10 cm. Then, the AM was placed over an impregnated dressing support, fixing the ends with sterile silk sutures. The membrane was introduced into an ethyl vinyl acetate cryopreservation bag (Macopharma, Madrid, Spain) with a cryoprotectant solution made of 15% dimethyl sulfoxide (WAK-Chemie Medical, GmbH), 70% culture medium (TC199, Thermo Fisher Scientific, Rockford, IL, United States of America) and 15% human serum albumin (Grifols, Barcelona, Spain) and then frozen at −80°C.

**FIGURE 1:**
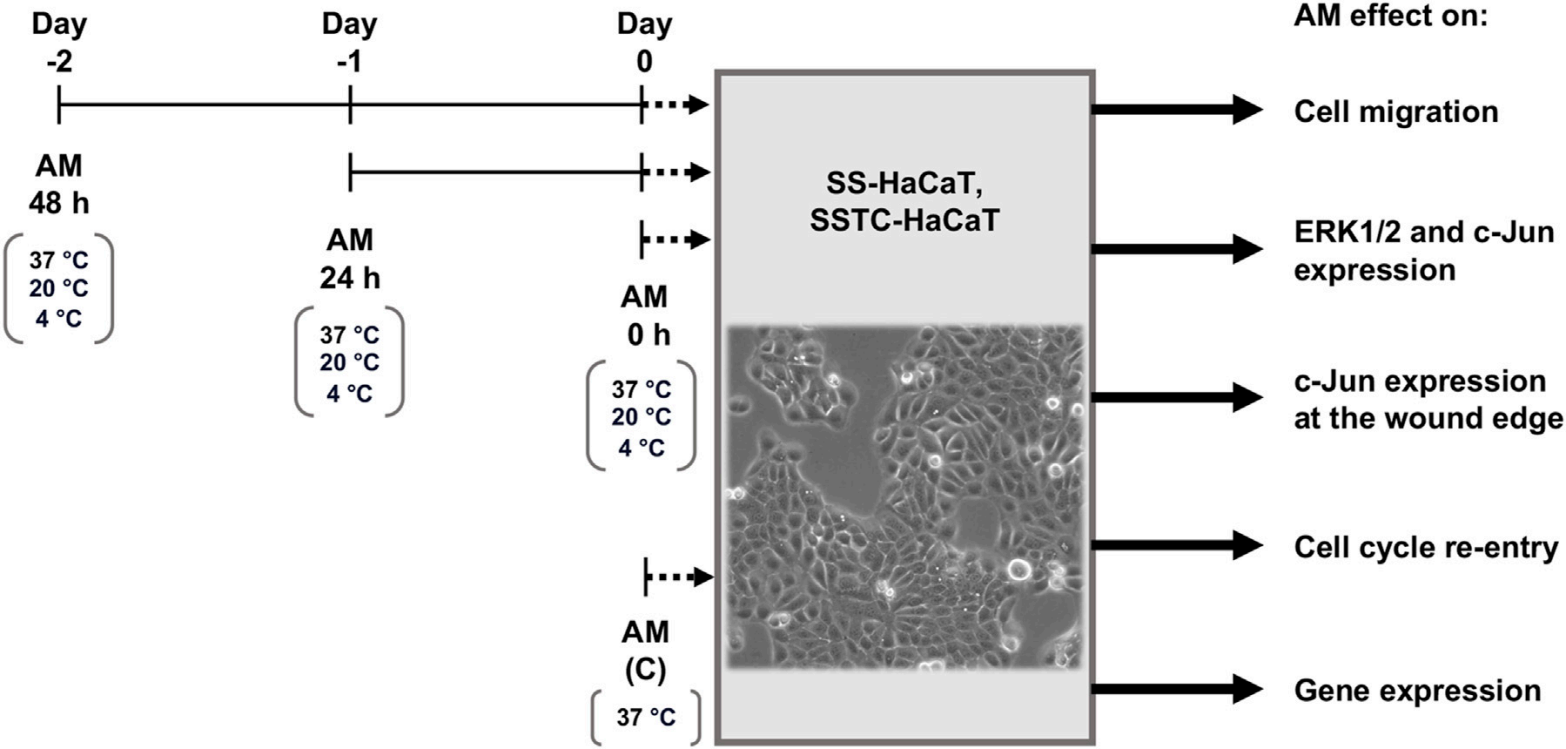
Incubation time and conditions after amniotic membrane freeze–thawing cycle. Amniotic membrane (AM) pieces were taken from liquid nitrogen, thawed, and washed in either FBS-free DMEM or physiological saline solution (PSS). Three incubation periods of AM pieces were performed before their addition to serum-starved HaCaT (SS-HaCaT) cells and serum-starved-TGF-β1-chronified HaCaT (SSTC-HaCaT) cells. These pieces were maintained in DMEM or PSS under different temperatures: 37°C, 20°C, or 4°C. After AM stimulation on SS-HaCaT and SSTC-HaCaT cells, several assays were carried out with these cells to study the effect of AM on cell migration, key-protein activation, subcellular localization, cell cycle re-entry, or key-gene expression.

For the subsequent assays with HaCaT cells, different treatments were carried out for AM pieces, as shown in Figure 1. For routine assays with HaCaT cells, the standard protocol for AM activation was thawing the pieces at 37°C and washing them three times with physiological saline solution to remove cryoprotectants. After this, AM pieces were incubated in the same media for 2 h at 37°C in a 7.5% CO_2_ humified atmosphere (Figure 1, AM (C)) as previously described (Bernabe-Garcia et al., 2017; Ruiz-Canada et al., 2018; Ruiz-Canada et al., 2021; Castellanos et al., 2017). In order to challenge thawed AM activity and potential through time, different AM pieces were incubated for 48 h (Figure 1, AM 48 h), 24 h (Figure 1, AM 24 h), or 2 h standard time (Figure 1, AM 0 h) before their application on HaCaT cells. Additionally, AM activity was challenged by subjecting AM pieces to three different temperatures (37°C, 20°C, or 4°C) and different incubation media (FBS-free DMEM or physiological saline solution, PSS). Furthermore, an additional preservation medium was made by adding human serum albumin at 4% final concentration to PSS (PSS + A). The conditions used in each assay are indicated in figure legends or the results section.

### Wound healing scratch assay

HaCaT cells were seeded in 24-well plates until they reached 100% confluence in complete DMEM. At that point, the medium was changed to FBS-free medium for 24 h. After this time, a cross-shaped scratch was made on the serum-starved HaCaT monolayer using a sterile p-200 µL pipette tip (time 0 h). After replacing the FBS-free medium to wash out released cells and cell debris, thawed AM pieces were added to wells at two AM pieces per well, floating in the cell culture above the scratched epithelium.

In parallel, a positive control was set by adding 10 ng/mL epidermal growth factor (EGF, Sigma-Aldrich, St Louis, MO, United States of America). Additionally, a pharmacological inhibitor against epidermal growth factor receptor (EGFR), PD153035, was added at 2.5 µM (Bos et al., 1997; Bennett et al., 2001; Shang et al., 2016) to set a negative control. After a 24-h incubation period, cells were fixed with 4% formaldehyde (Applichem GmbH, Darmstadt, Germany) in phosphate-buffered saline solution (PBS, Biowest, Nuaillé, France) for 10 min. Finally, well plates were washed twice with PBS. Pictures were taken at ×10 magnification using an Axiovert 5 ZEISS optical microscope with a digital camera (ZEISS, Jena, Germany). To quantify cell migration, the areas of the gaps in the wounds were measured by ImageJ software. The initial cell-free surface (time 0 h) was subtracted from the endpoint cell-free surface (time 24 h) and plotted in a graph (Liarte et al., 2018).

### Amniotic membrane cell integrity assay

AM pieces incubated in PSS at 4°C for 0 h, 24 h, and 48 h were placed on glass slides and stained *in situ* with exclusion stain trypan blue to reveal death cells in the AM tissue. Then, pictures were taken at ×10 magnification using an optical microscope equipped with a digital camera (Motic Optic AE31, Motic Spain, Barcelona, Spain). To quantify cell integrity, the pictures were analyzed by ImageJ software by counting viable cells and total cells.

### Cell-front migration assay and subcellular localization assay by immunofluorescence

HaCaT cells were grown until they reached 100% confluence on round glass-coverslips in complete DMEM placed on 6-cm diameter plates. At this time, cells were washed, and a 24-h serum-starvation period was set by replacing the medium with FBS-free DMEM. Then, the epithelium of SS-HaCaT cells was scratched using a razor blade to open a gap big enough to follow the migration of cells for 24 h, and the scratched epithelium was returned to culturing plates with FBS-free DMEM. The new wound was set as time 0 h of the experiment, and then AM pieces were added, which remained floating in the cell culture above the wound. After the selected incubation times, glass-coverslips were fixed with 4% formaldehyde in PBS for 10 min and washed twice with PBS. Immunostaining was done as in Stelling-Ferez et al. (2023). Briefly, cells were permeabilized with 0.3% Triton X-100 (Sigma-Aldrich, St Louis, MO, United States of America) in PBS for 15 min followed by blocking for 30 min at room temperature in PBS solution with 10% FBS, 5% skim milk (Beckton Dickinson, Franklin Lakes, NJ, United States of America), 0.3% bovine serum albumin (BSA, Sigma-Aldrich, St Louis, MO, United States of America), and 0.1% Triton X-100. Then, samples were incubated for 1 h at room temperature with anti-phospho-histone H3 or anti-c-Jun antibodies, diluted in blocking solution without skim milk. Proper fluorescent-labeled secondary antibodies (see the Antibodies section) were co-incubated for 30 min with Alexa Fluor 594-conjugated phalloidin (Molecular Probes, Thermo Fisher Scientific, Waltham, MA, United States of America) and Hoechst 33258 (Fluka, Biochemika, Sigma-Aldrich, St Louis, MO, United States of America) to reveal actin cytoskeletons and nuclei, respectively. Once samples were immunostained, and image acquisition was performed with a confocal microscope (LSM 510 META from ZEISS, Jena, Germany). The setting of images was performed using ZEISS Efficient Navigation (ZEN) interface software (ZEISS, Jena, Germany). When a wider view of the migration front was required, concretely in c-Jun staining (indicated in Figures), 4 × 3 linked fields were acquired with the “Tile scan” ZEN tool.

The quantification of c-Jun levels in immunofluorescence pictures was done as in Stelling-Férez et al. (2023). Briefly, images were analyzed and quantified using ImageJ software. For this purpose, pictures in 8-bit three-channel format (Red, Green, Blue, RGB) were divided into four separate color channels (three pictures). Using the blue channel picture (Hoechst staining), regions of interest (ROIs) were established to define each nucleus, creating as many ROI masks as nuclei in the image. Then, by overlapping these masks onto the corresponding green channel picture (c-Jun staining), we calculated the green intensity value of each nucleus (ROI). Because of the large area covered in each picture (Tile scan), they were divided into four equal sectors (S1, S2, S3, and S4), with S1 being the outermost edge of the wound. Within each sector, the quantified signal of each nucleus was used as a replicate to obtain c-Jun intensity data in each of the performed conditions. Regarding phospho-histone H3 levels, immunofluorescence pictures were divided into three equal sectors (S1, S2, and S3), and the quantification in each sector was performed by counting positive p-H3 cell nuclei.

### Western blot

HaCaT cells were seeded and allowed to reach 60%–70% confluence in 6-cm-diameter plates. At this time, complete DMEM was replaced by FBS-free DMEM, incubating the cells for a 24-h period. Then, serum-starved HaCaT cells were treated with AM pieces incubated in PSS at 4°C for 0 h, 24 h, and 48 h. After the incubation times (indicated in the figure), cells were harvested, washed twice with ice-cold PBS, and lysed with 20 mM TRIS pH 7.5, 150 mM NaCl, 1 mM EDTA, 1.2 mM MgCl2, 0.5%, Nonidet p-40, 1 mM DTT, 25 mM NaF, and 25 mM β-glycerophosphate supplemented with phosphatase inhibitors (I and II) and protease inhibitors (all from Sigma-Aldrich, St Louis, MO, United States of America). The total protein amount was measured and normalized by Bradford assay (Ernst and Zor, 2010) (Sigma-Aldrich, St Louis, MO, United States of America). The extracts were analyzed by SDS-PAGE, together with a molecular weight marker used as standard, Precision Plus Dual Color (Bio-Rad, Hercules, Ca, United States of America). Next, Western blot (WB) was assayed using the indicated antibodies. Blots were revealed by using horseradish peroxidase substrate (ECL) (GE Healthcare, GE, Little Chalfont, United Kingdom), and images were taken with a Chemidoc XRS1 (Bio-Rad, Hercules, Ca, United States of America). Western blot bands were quantified as in Stelling-Ferez et al. (2022). Mainly, Western blot pictures in 8-bit format were processed in ImageJ software. Subsequently, in all Western blot pictures, a lane was established for each of the samples. In each lane, the band was selected according to the specific size (kDa) of the protein of interest. For each total protein and its phosphorylated version, each band’s intensity peak was plotted, and next, the area under the plot was measured by using the “Wand (tracing) tool” of ImageJ to obtain the intensity value. In order to normalize, obtained intensity values were referred to obtained intensity values of either the unphosphorylated form of the protein (total) or a loading control protein such as β-actin when the unphosphorylated form was undetectable (non-available antibody for detecting the unphosphorylated form).

### Real-time PCR

HaCaT cells were chronified as described above by seeding them in 6-cm-diameter plates. Then, SSTC-HaCaT cells were treated with AM pieces incubated in PSS at 4°C for 0 h, 24 h, and 48 h. The procedure was done as in Liarte et al. (2023). Essentially, total RNA was extracted and purified using the RNeasy-mini kit system (Qiagen, Venlo, Netherlands). Then, 800–900 ng of RNA from independent samples were retro-transcribed using iScript reagents (Bio-Rad, Hercules, CA, United States of America). The resulting cDNA was used for quantitative PCR (qPCR) by using a SYBR premix ex Taq kit (Takara Bio Europe/Clontech, Saint-Germain-en-Laye, France), following the manufacturer’s instructions. For each mRNA, gene expression levels were normalized to the glyceraldehyde 3-phosphate dehydrogenase (GAPDH) content of each sample by applying the comparative Cq method (2−∆∆Cq). The primers used are detailed in Table 1. Replicates from three independent experiments were quantified. Analyzed data represent the mean ± SEM.

**TABLE 1:** Different primers used to study the expression of several genes. *GAPDH*, glyceraldehyde-3-phosphate dehydrogenase; *CDKN2B*, cyclin-dependent kinase inhibitor 2B; *CDKN1A*, cyclin-dependent kinase inhibitor 1A; *CCNA2*, A2-type cyclin; *IL6*, interleukin 6; *GLB1*, galactosidase beta 1; *FUCA1*, alpha-L-fucosidase 1.

Primer name	Primer sequence 5′ to 3′
*GAPDH*-Fwd	ACC​ACA​GTC​CAT​GCC​ATC​AC
*GAPDH*-Rev	TCC​ACC​ACC​CTG​TTG​CTG​TA
*CDKN2B*-Fwd	ATGCGCGAGGAGAACAAG
*CDKN2B*-Rev	CTC​CCG​AAA​CGG​TTG​ACT​C
*CDKN1A*-Fwd	ATG​TCA​GAA​CCG​GCT​GGG​GAT​G
*CDKN1A*-Rev	GGG​CTT​CCT​CTT​GGA​GAA​GAT​C
*CCNA2*	Proprietary sequence (Sigma KiCqStart)
*IL6*	Proprietary sequence (Sigma KiCqStart)
*GLB1*	Proprietary sequence (Sigma KiCqStart)
*FUCA1*-Fwd	AGT​CAC​CCT​GTT​GCC​TAT​GG
*FUCA1*-Rev	TTT​GGC​GCT​TTT​AGA​TTG​CT

### Cell cycle re-entry

HaCaT cells were grown until they reached 60% confluence on 12-well plates. At this point, the medium was replaced with FBS-free DMEM. HaCaT cells were grown in serum-deprived DMEM with or without TGF-β1 (Liarte et al., 2020). The produced SS- and SSTC-HaCaT cells were treated with AM pieces incubated in PSS at 4°C for 0 h, 24 h, and 48 h. After 27 h treatment, SS- and SSTC-HaCaT cells were harvested by detachment with 0.25% trypsin/EDTA (Biowest, Nuaillé, France). For cell cycle analysis, samples from detached cells were centrifuged, and the resulting pellet was immediately fixed with cold 70% ethanol in PBS and stored at 4°C for 30 min. Subsequently, cells were centrifuged at 1,000 rpm for 10 min and resuspended in PBS. Finally, the cells were treated with a solution of 20 μg/mL ribonuclease A and 400 μg/mL propidium iodide (all from Sigma-Aldrich, St. Louis, MO, United States of America) in PBS. Cells were analyzed through flow cytometry using a BD LSRFortessa™ X-20 Cell Analyzer (BD Biosciences, Pharmingen, Beckton Dickinson, Franklin Lakes, NJ, United States of America).

### Antibodies

The following commercial primary antibodies were used: anti-phospho-ERK1/2 (1:2000 in 1% BSA), anti ERK1/2 (1:1,000 in 1% BSA), anti-phospho-c-Jun (1:1,000 in 1% BSA), and anti-c-Jun (1:1,000 in 1% BSA) (all from Cell Signaling Technology, Danvers, MA, United States of America); anti-phospho-histone H3 (Santa Cruz Biotechnology, Heidelberg, Germany); and anti-β-actin (Sigma-Aldrich, St Louis, MO, United States of America). Secondary antibodies were anti-rabbit IgG horseradish peroxidase linked F(ab')2 I fragment (from donkey) (GE Healthcare, GE, Little Chalfont, United Kingdom), anti-mouse IgG_1_ (BD Pharmingen, Beckton Dickinson, Franklin Lakes, NJ, United States of America), and Alexa Fluor 488 conjugated anti-mouse (from donkey) (Thermo Fisher Scientific, Rockford, IL, United States of America).

### Statistical analysis

The collected data were analyzed using GraphPad Prism 7 software. The standard error and mean were calculated for every analysis, and statistical tests were performed with a 95% confidence interval; consequently, *p*-values lower than 0.05 were considered statistically significant. In the figure legends, the asterisks denote statistically significant differences between conditions (*p < 0.05, **p < 0.005, ***p < 0.001, and ****p < 0.0001). The data of intensity values from Western blots were analyzed by one-way ANOVA, comparing the mean of each condition with the mean of every other condition. In this, a Tukey’s multiple comparisons test was performed. Data of intensity values obtained from c-Jun and p-H3 nuclei quantifications in immunofluorescence pictures were analyzed by two-way ANOVA. A two-way ANOVA analysis, followed by a Tukey’s multiple comparison test, was performed to compare the p-c-Jun or p-H3 intensity mean between sectors from different conditions (e.g., S1 Control versus S1 AM 0 h).

## Results

### AM preserved at room temperature is significantly powerful for HaCaT cell migration

We have previously shown that AM significantly stimulates HaCaT keratinocyte migration in wound-healing scratch assays (Alcaraz et al., 2015; Bernabe-Garcia et al., 2017). In order to challenge the therapeutic properties of AM, thawed AM pieces were incubated for different times in different carrying solutions and at different temperatures before testing their therapeutic potential by different cell assays (for a more detailed description, see the Materials and methods section) (Figure 1). To assess the activity of AM on cell migration, serum-starved HaCaT cells were stimulated with pieces of AM maintained at 20°C (room temperature) in FBS-free DMEM after thawing. That was outside the incubator in a 0.04% CO_2_ atmosphere. In parallel, AM pieces thawed and activated at 37°C, 7.5% CO_2_ in DMEM (AM (C) 0, AM 24 h) were added to SS-HaCaT cells.

**FIGURE 2:**
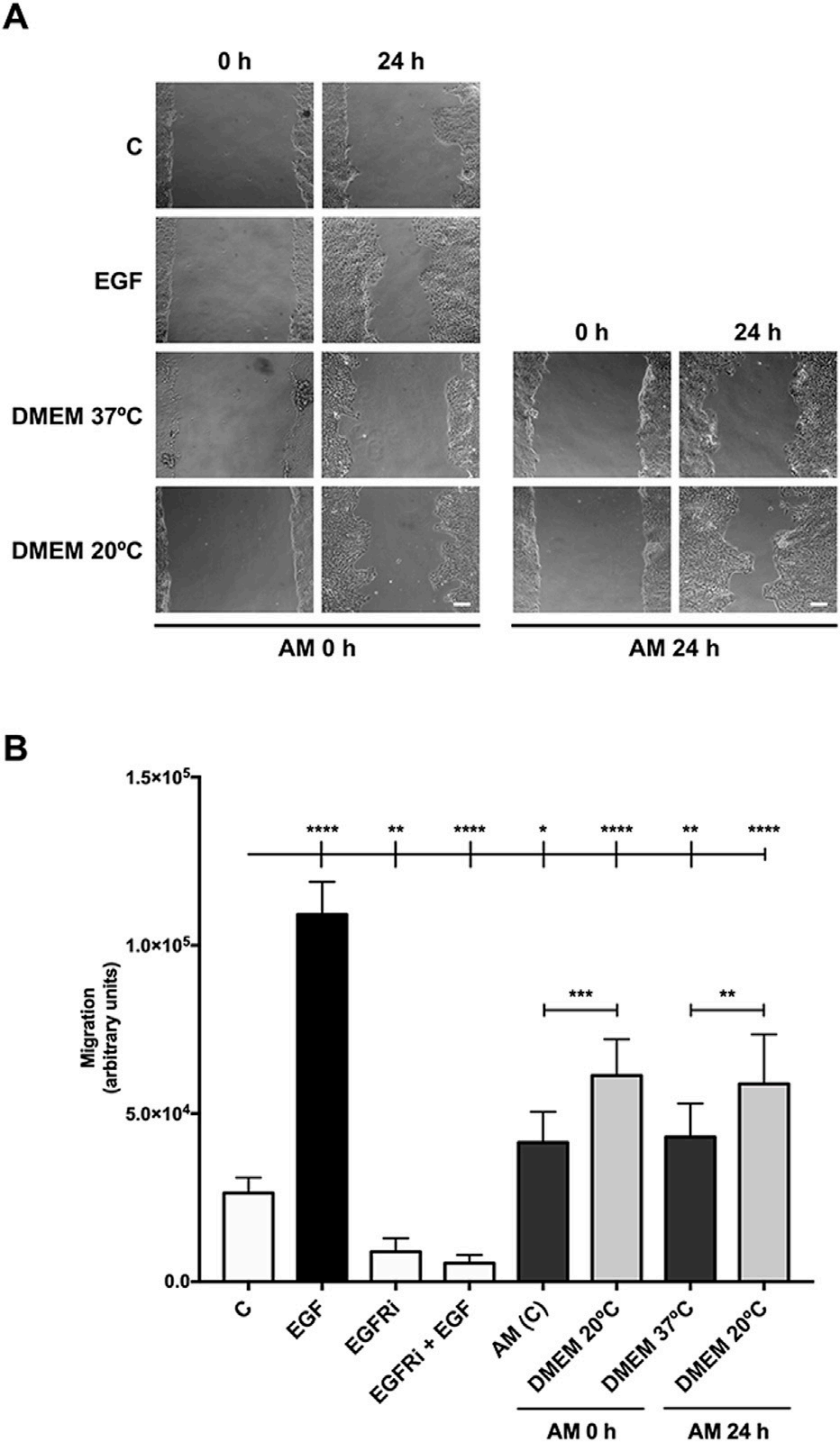
Amniotic membrane kept at room temperature in the cell culture medium showed powerful effects on HaCaT cell migration. Confluent SS-HaCaT cells were scratched with a pipette tip, treated with AM, and allowed to migrate for 24 h. **(A)** Representative images of the assay showing basal cell migration (C) compared to those with thawed and incubated AM for 0 h and 24 h in FBS-free DMEM at either 37°C or 20°C (AM 0 h and AM 24 h). Standard protocol for AM was performed in parallel (AM (C)). EGF (10 ng/mL) was added as a positive control. A pharmacological inhibitor against EGFR, PD153035 (EGFRi), was added as a negative control. The scale bar indicates 50 µm. **(B)** The bar graph represents migration as the difference between gap areas at time 0 h and time 24 h in each condition, defined as migration. Asterisks indicate statistically significant differences between conditions according to a one-way ANOVA statistical analysis (*p < 0.05, **p < 0.005, and ****p < 0.0001).

Interestingly, all these AM pieces, activated for 2 h (0 h) and 24 h, promoted cell migration from the wound edges in the scratch assay (Figure 2A). When migration was quantified, AM pieces maintained at 20°C significantly increased SS-HaCaT cell migration with more potency than pieces maintained at 37°C (Figure 2B). These results suggest that the specific conditions of a cell culture incubator are not critical to maintain AM therapeutic potency because room temperature and a lower content of CO_2_ in the atmosphere both did not affect AM migratory effect and, remarkably, seemed to better maintain the benefits of AM on the migration of serum-starved HaCaT cells.

### AM kept at 4°C maintains its cell integrity and promotes HaCaT cell migration

**FIGURE 3:**
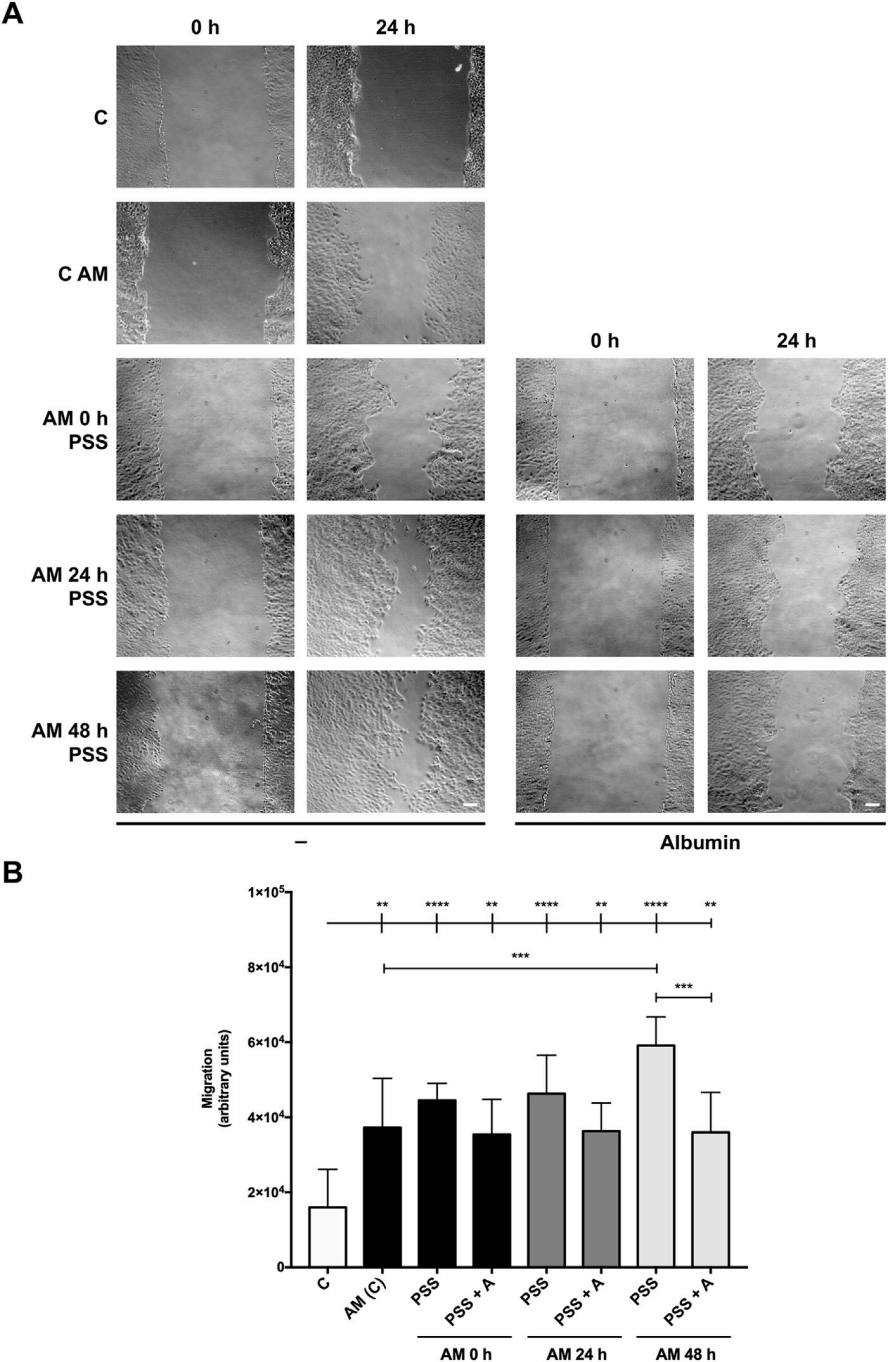
Amniotic membrane kept in PSS remains active even after 48 h. Confluent serum-starved HaCaT cells were scratched with a pipette tip, treated with AM, and allowed to migrate for 24 h. **(A)** Representative images of the assay showing basal cell migration (C) compared to those with thawed and incubated AM for 0 h, 24 h, and 48 h in PSS or PSS +4% albumin (PSS + A) at 4°C. Standard protocol for AM was performed in parallel (AM (C)). The scale bar indicates 50 µm. **(B)** The bar graph represents migration as the difference between gap areas at time 0 h and time 24 h in each condition, defined as migration. Asterisks indicate statistically significant differences between conditions according to a one-way ANOVA statistical analysis (**p < 0.005, ***p < 0.001, and ****p < 0.0001).

With the previous results obtained in scratch assays, we challenged AM incubation at cooler temperatures and longer times, considering its plausible delivery to distant healthcare facilities. Therefore, AM pieces were kept in physiological saline solution, as many biologic reagents are maintained and delivered using this medium (Blumberg et al., 2018; El-Amawy and Sarsik, 2021). In addition, we subjected AM pieces to a colder temperature, 4°C in PSS, after the freeze–thawing cycle, for 0 h, 24 h, and 48 h, which may be beneficial for the preservation of AM therapeutic properties. Then, serum-starved HaCaT cells were stimulated. Additionally, AM fragments were kept under the same conditions with the addition of human albumin (Figure 3), a colloid used to preserve many biologic solutions (Bihari et al., 1985), and their potency was compared to standard AM at 37°C. Strikingly, AM in PSS at 4°C was able to significantly increase HaCaT cell migration for all incubation time periods, 0 h, 24 h, and 48 h (Figure 3A). Additionally, AM pieces maintained in PSS showed cell migration percentages similar to control conditions (C), with a tendency to be higher than standard AM at 37°C (AM (C)), with special significance at time 48 h (Figure 3B). In contrast, AM pieces maintained in PSS with albumin (PSS + A) exerted a slightly lower potency on cell migration than AM kept in PSS alone. Additionally, a cell integrity test was performed using fragments of AM from the same experiment before the stimulation of HaCaT cells (Supplementary Figure S1). AM pieces were studied to quantify epithelial cell integrity, and surprisingly, no statistical differences were found between the different conditions. In fact, even at the longest 4°C time period (48 h in PSS), only a slight decrease was noticed with no statistical significance.

All these data suggest that maintaining AM in PSS at 4°C keeps most of its cells’ integrity and potency for cell migration, with a slight amelioration in the absence of added albumin in the preserving solution.

### Amniotic membrane kept at 4°C in physiological saline solution induces MAP kinase ERK and c-Jun activation

**FIGURE 4:**
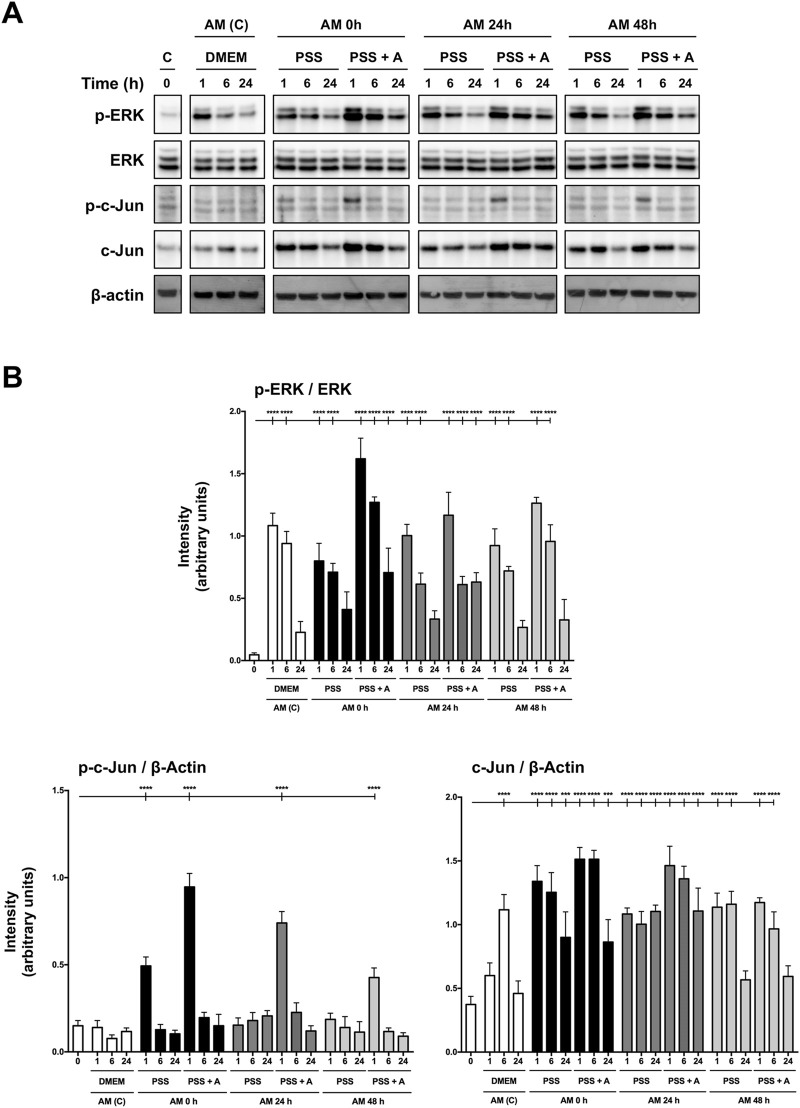
Amniotic membrane maintained in saline solution stimulates phosphorylation of both ERK and c-Jun, necessary for HaCaT cell migration. **(A)** Total protein extracts from sub-confluent serum-starved HaCaT cells treated with AM for 1 h, 6 h, or 24 h. AM had been incubated for 0 h, 24 h, or 48 h in PSS at 4°C. Standard protocol for AM was performed in parallel (AM (C)). Protein extracts were assayed by targeting phospho-ERK1/2, ERK1/2, phospho-c-Jun, and c-Jun. β-actin was used as a loading control. A representative experiment is shown. **(B)** The bar graphs represent the intensity values of each protein assayed by Western blot. The intensity values were quantified by ImageJ software and collated. P-ERK1/2 values were normalized to their total form with ERK. P-c-Jun and c-Jun values were normalized with β-actin. Asterisks indicate statistically significant differences between the selected conditions according to a one-way ANOVA statistical analysis (****p < 0.0001).

In recent publications, we have shown that AM promotes HaCaT cell migration by inducing MAP kinases and c-Jun phosphorylation, key regulatory proteins that drive cell migration and proliferation (Alcaraz et al., 2015; Ruiz-Canada et al., 2018). The results obtained from cell migration encouraged us to use the 4°C preservation temperature as the standard to carry on with the rest of the experiments, as the provided migration is one of the key therapeutic effects of AM on chronic wounds (Insausti et al., 2010; Alcaraz et al., 2015). All samples of AM kept at 4°C for different periods of time were tested on serum-starved HaCaT cells. In all circumstances, the pieces clearly induced the activation by phosphorylation of both ERK1/2 and c-Jun (Figure 4A). In addition, AM kept in PSS at 4°C promoted c-Jun overexpression for all the incubation times tested (Figure 4A). When quantified, different activation patterns were observed between AM preserved in PSS and in PSS plus albumin. In the case of phopsho-ERK1/2, AM in PSS showed very similar phosphorylation levels to standard AM (AM (C), Figure 4B), without statistical differences. However, AM preserved in PSS + albumin exhibited higher phospho-ERK1/2 and phospho c-Jun levels than AM in PSS only. Lastly, c-Jun total form showed significant increases with either AM kept in PSS or AM in PSS + albumin, in a more efficient fashion than with standard membrane (AM (C)). All these data suggest that AM kept in PSS at 4°C clearly enhances MAP kinases and c-Jun transcription factor activation, with a slight improvement in fragments that were kept in PSS with albumin. This is reinforced and compatible with the results observed in wound healing scratch assays.

### Amniotic membrane kept at 4°C in physiological saline solution promotes c-Jun overexpression at wound-edge cells

**FIGURE 5:**
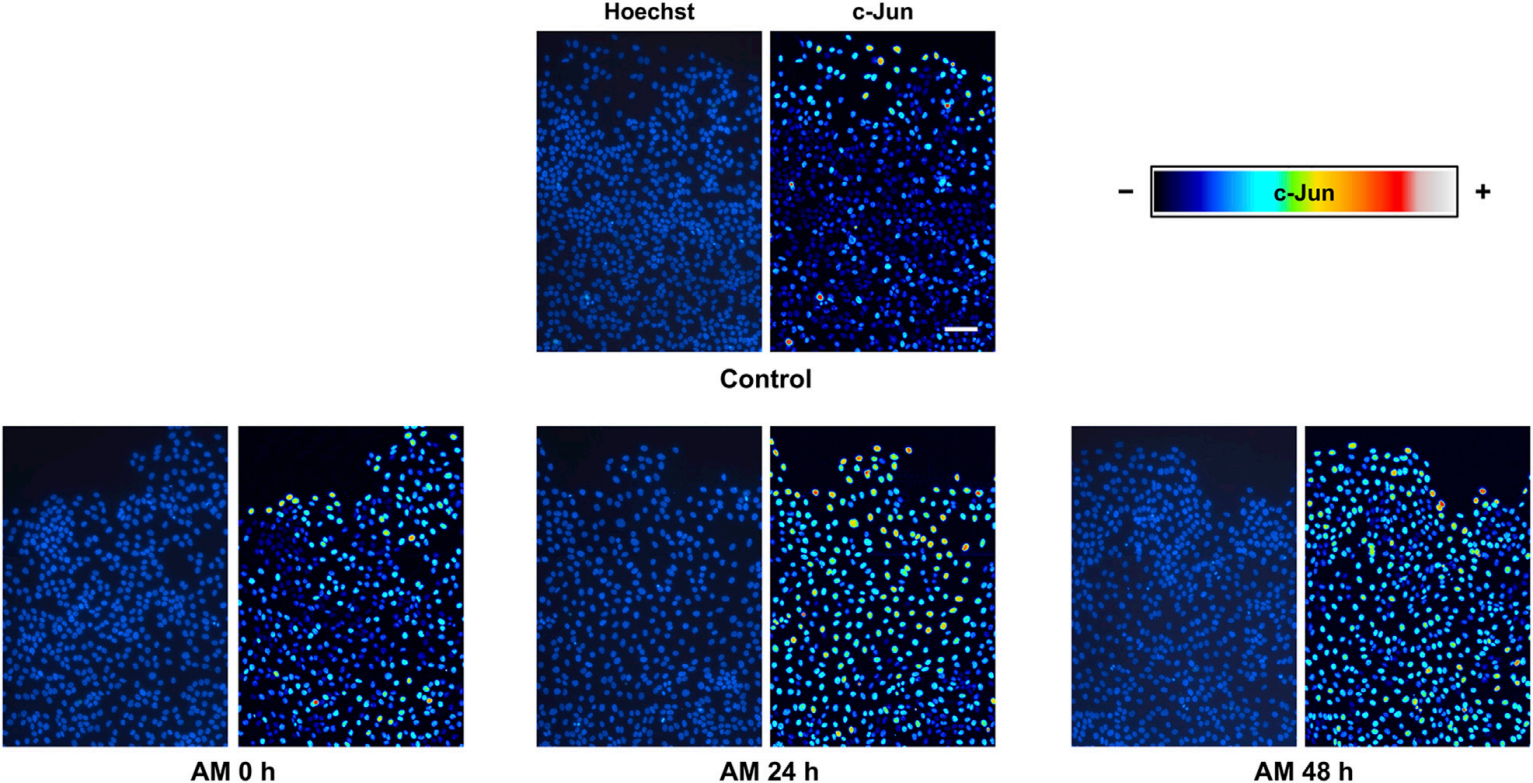
AM kept at 4°C in physiological saline solution promotes c-Jun overexpression in serum-starved HaCaT cells at the wound edge. Confluent SS-HaCaT cells were scratched and treated with AM and allowed to migrate for 24 h. AM had been incubated for 0 h, 24 h, or 48 h at 4°C in PSS. Cells were immunostained with a specific antibody against c-Jun. Co-staining with Hoechst-33258 was used to reveal nuclei. C-Jun intensity values were transformed using a pseudo color scale, a rainbow, for better visualization. Cell nuclei are shown in blue. Images were obtained with a confocal microscope at ×40 magnification. This experiment was repeated at least three times. The scale bar indicates 60 µm.

It has been shown that a wounded epithelium expresses and phosphorylates c-Jun transcription factor at the wound edge, an event that is critical for cell migration to promote reepithelization (Herdegen et al., 1997; Li et al., 2003). Indeed, this stimulation is increased with AM and even with bioactive compounds to enhance cell migration, consequently promoting wound healing (Alcaraz et al., 2015; Ruiz-Canada et al., 2018; Stelling-Ferez et al., 2022; Stelling-Férez et al., 2023). We focused on serum-starved HaCaT cell fronts to study c-Jun expression at cell nuclei in response to AM kept in PSS at 4°C. After 24-h stimulation, we observed a clear overexpression of c-Jun in response to AM kept in PSS for any of the studied time periods, especially at the nuclei of cells at the wound edge (Figure 5). The quantification of c-Jun staining in cell-front pictures revealed a significant increase of c-Jun in response to AM 0 h compared to control conditions (Supplementary Figure S2). Interestingly, c-Jun overexpression was higher as cells were closer to the wounded area, at the leading edge, with deeper maintenance of c-Jun overexpression in sectors S2, S3, and S4 than in the control sample. Indeed, this increase of c-Jun in serum-starved HaCaT cells implied higher cell recruitment in response to all AM kept in PSS than to the control. Strikingly, AM induction on c-Jun expression was not diminished by long preservation times of 24 h and 48 h. It also exhibited more nuclei with overexpressed c-Jun, an effect that was more noticeable at 24 h. In summary, AM pieces preserved in PSS at 4°C, regardless of the preservation time, were able to promote c-Jun overexpression at the wound edge.

### Amniotic membrane kept at 4°C in physiological saline solution increases proliferation at wound-edge cells

**FIGURE 6:**
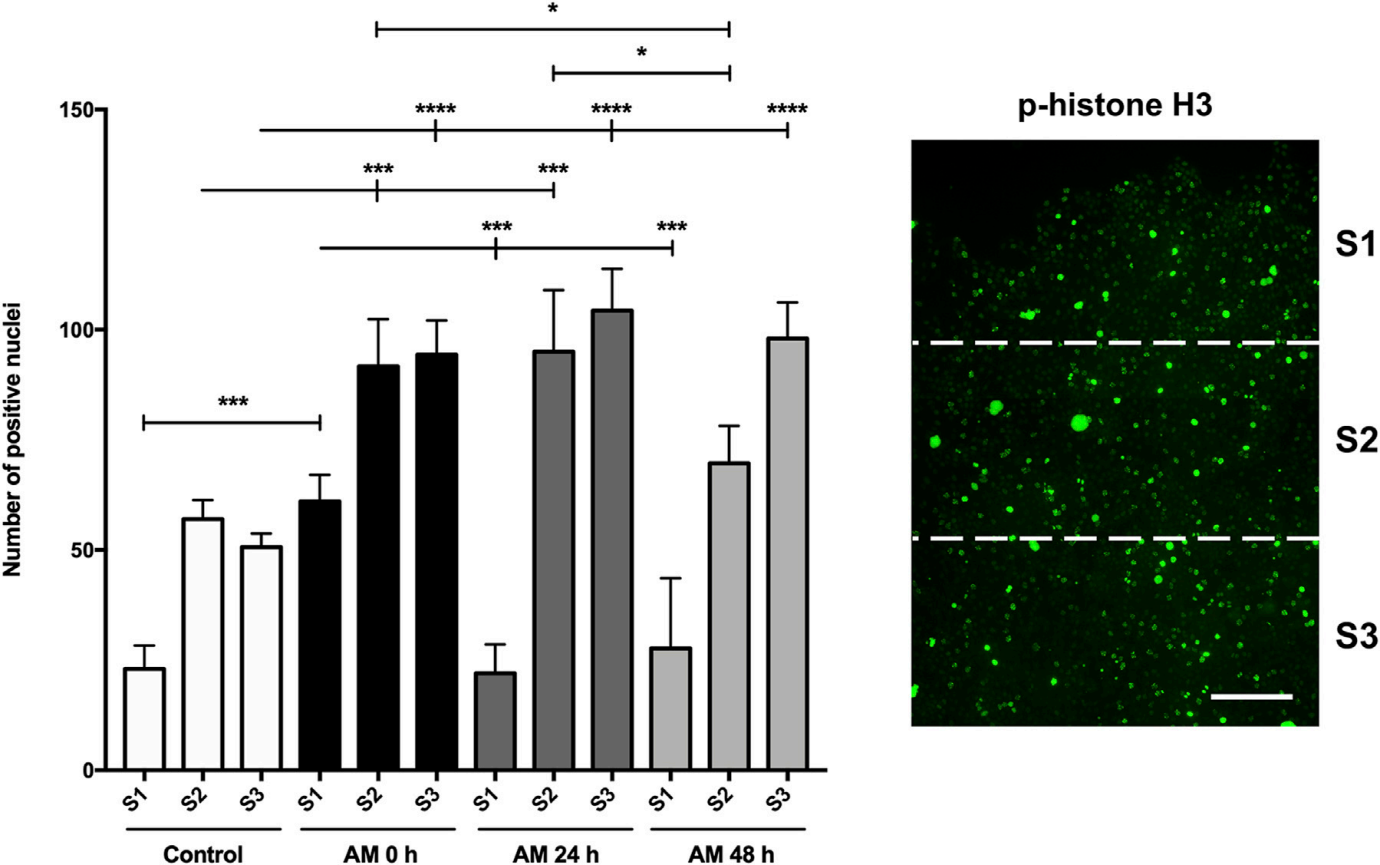
AM kept at 4°C in PSS increases phosphorylation of histone H3 at cells behind the wound edge. Confluent SS-HaCaT cells were scratched and treated with AM, allowing them to migrate for 24 h. AM had been incubated for 0 h, 24 h, or 48 h at 4°C in PSS. Cells were immunostained with a specific antibody against phospho-histone H3. The bar graph indicates the number of positive p-histone H3 nuclei along the cell front and the artificial wound, equally divided into three sectors. The scale bar indicates 80 µm. Asterisks indicate statistically significant differences between the selected conditions according to a two-way ANOVA statistical analysis followed by Tukey’s multiple comparisons test (*p < 0.05, **p < 0.005, ***p < 0.001, and ****p < 0.0001).

During wound healing, both keratinocyte migration and proliferation are critical for restoring the epidermis and wound closure (Rodrigues et al., 2019; Reinke and Sorg, 2012; Calabrese et al., 2022). Histone H3 is phosphorylated and becomes p-histone H3 during cell division (Paluch et al., 2016; Yuceer and Baspinar, 2024). In a previous article, we showed that AM treatment increases the phosphorylation of histone H3 on scratched HaCaT monolayers, which indicated a promotion of proliferation (Liarte et al., 2023). We used different AM kept in PSS for different times to stimulate scratched fronts of serum-starved HaCaT cells. The analysis of positive histone H3 phosphorylation-activated cells showed that AM in PSS, for all the times tested (0 h, 24 h, and 48 h), was capable of increasing the number of positive cells, thus resulting in a significantly higher number of dividing active cells than the control unstimulated sample (Figure 6; Supplementary Figure S3). It is worth noting that these increases were significant when comparing sectors 2 and 3, located further away from the artificial wound edge (Figure 6). Strikingly, no significant differences were reached between the different AM incubation times in PSS at 4°C, except for a significant increase that was noticeable at 0 h in sector 1.

These data suggested that AM induced the proliferation of cells at the wound edge of an artificial wound regardless of the time for which the amniotic membrane was kept in PSS at 4°C after thawing.

### Amniotic membrane kept at 4°C in saline solution rescues HaCaT cells from TGF-β-induced cell cycle arrest

**FIGURE 7:**
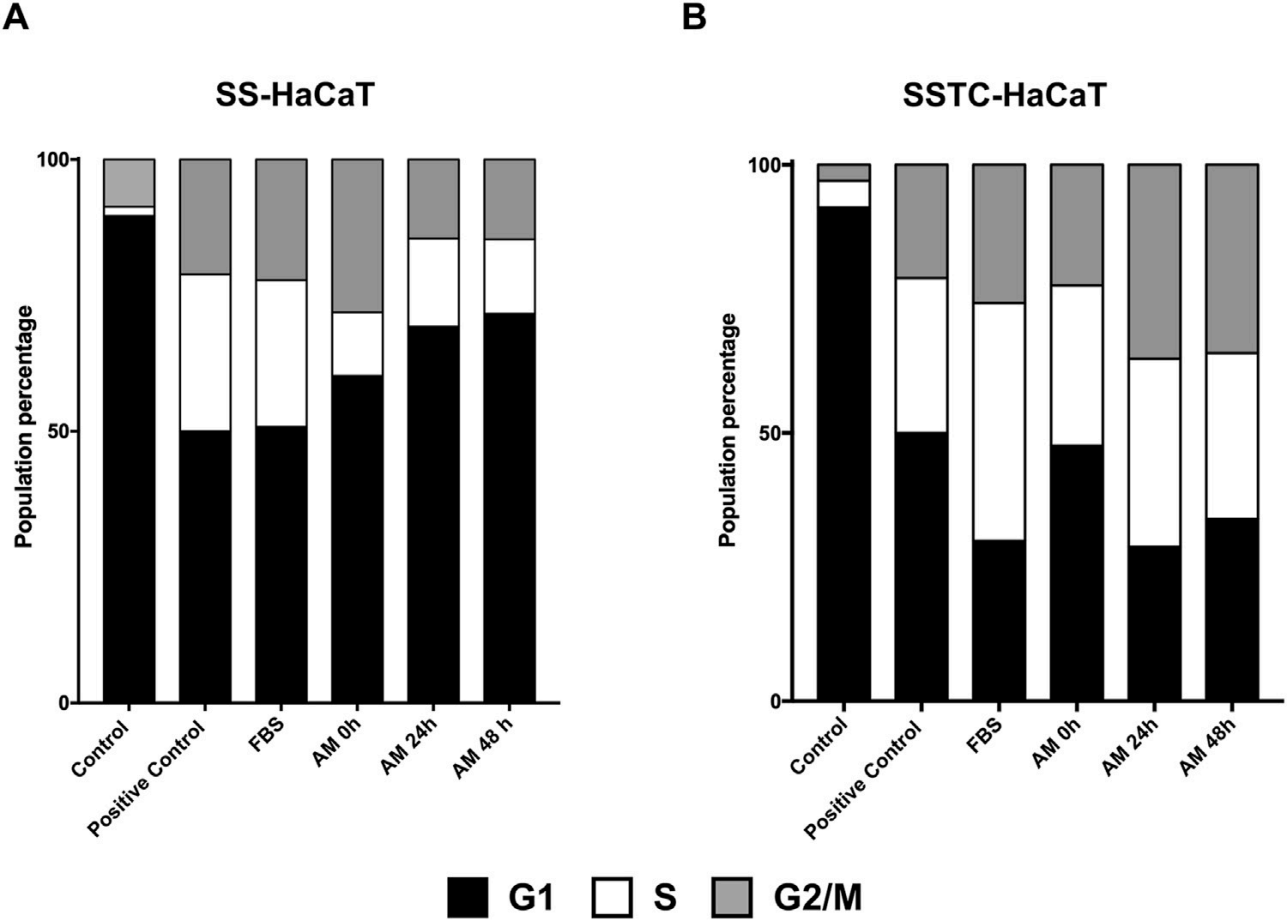
AM kept at 4°C in PSS rescues HaCaT cell cycle arrest. Both SS- and SSTC-HaCaT cells were analyzed after 24 h treatment with AM. AM had been incubated for 0 h, 24 h, or 48 h at 4°C in PSS. The experiment was repeated at least three times. Graphs represent the population distribution of SS-HaCaT **(A)** and SSTC-HaCaT cells **(B)** for each condition. Positive control indicates cells that had been growing in DMEM with serum (no growth restriction). FBS was added to a 10% final concentration in the media as a positive control.

AM promotes both cell migration and cell proliferation to enhance reepithelization and tissue regeneration (Toda et al., 2007; Alcaraz et al., 2015). The continuous stimulation of keratinocytes with TGF-β1 in the absence of serum produces modifications in HaCaT cells that resemble keratinocytes found in chronic wounds (Liarte et al., 2020). The consequence of HaCaT cell hyperstimulation with TGF-β1 has been named SSTC-HaCaT, and these cells show low migration rates and high expression of genes related to inflammation, senescence, and cell cycle arrest, producing their arrest in G1. However, many of these features can be rescued by applying AM. As a control for the SSTC-HaCaT cells, we used a similar treatment for 48 h in serum starvation but omitting TGF-β1. In this last case, the cells produced are named SS-HaCaT, and the affection in migration or proliferation/inflammation seemed normal compared to SSTC-HaCaT cells (Liarte et al., 2020; Liarte et al., 2023). SS-HaCaT cells stimulated for 24 h with membranes kept for 0 h, 24 h, or 48 h exhibited a clear rescue of cell cycle arrest when compared to unstimulated cells, which was similar to the effects of serum stimulation (Figure 7A). Strikingly, SSTC-HaCaT cells, characterized by a population dominated by G1 cells due to TGF-β1 pre-conditioning, were also rescued by all the conditions with AM in PSS at 4°C, in a similar fashion to SS-HaCaT cells (Figure 7B). In other words, neither the long incubation periods after thawing nor the cold temperature affected AM pro-proliferative effects either on SS-HaCaT or SSTC-HaCaT cells, resulting in a significant entrance in the cell cycle after AM stimulation.

### Amniotic membrane kept at 4°C in saline solution promotes a high cell proliferation expression profile in SSTC-HaCaT cells

**FIGURE 8:**
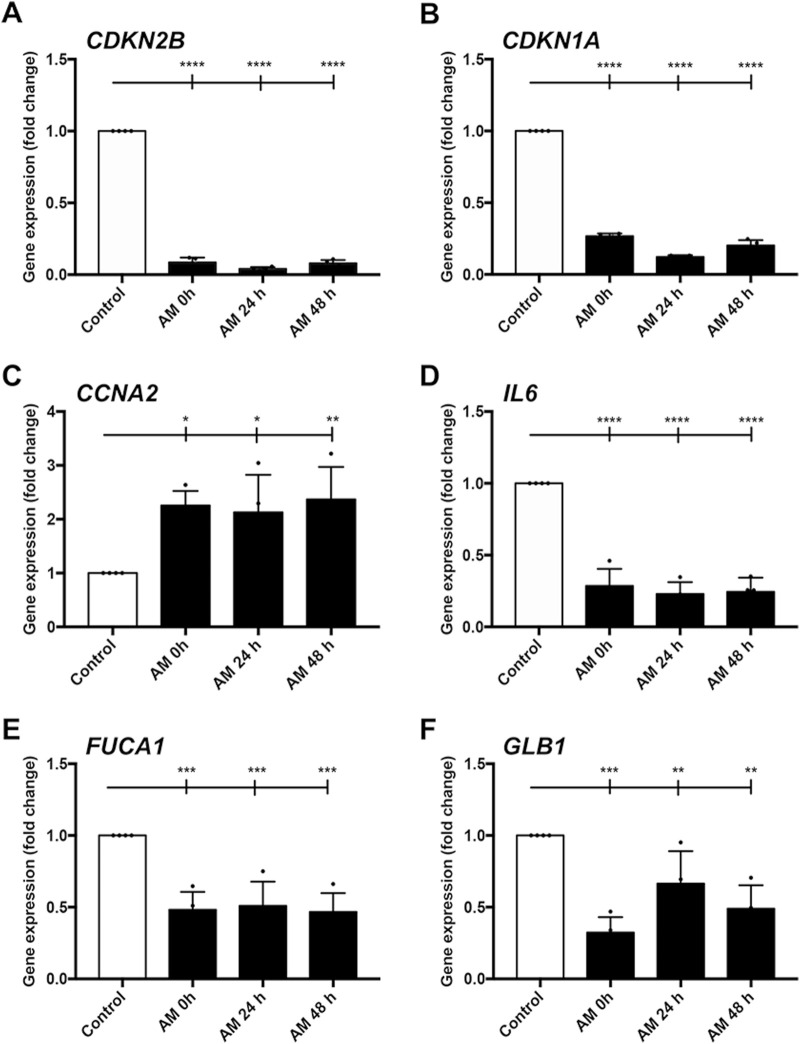
Amniotic membrane kept at 4°C in PSS restores SSTC-HaCaT cell chronified gene expression profile. Genes related to cell cycle, inflammation, and senescence were studied. Samples were obtained 24 h after AM treatment. AM had been incubated for 0 h, 24 h, or 48 h at 4°C in PSS. Bar graphs indicate expression level data, which are represented as fold change from the initial time. Genes studied were **(A)** *CDKN2B*; **(B)** *CDKN1A*; **(C)** *CCNA2*; **(D)** *IL6*; **(E)** *FUCA1*; **(F)** *GLB1*. Plots show the data of three technical replicates quantified by qPCR. Asterisks indicate statistically significant differences between the selected conditions according to a one-way ANOVA statistical analysis (*p < 0.05, **p < 0.005, ***p < 0.001, and ****p < 0.0001).

In line with AM rescue of cell proliferation, we continued to investigate AM proliferative and regenerative effects on HaCaT cells by studying the expression profiles of genes involved in cell growth, senescence, and inflammation. Previously, we deciphered that this perinatal tissue changes the entire HaCaT gene profile to overexpress genes related to proliferation and migration (Alcaraz et al., 2015). Moreover, SSTC-HaCaT cells are characterized by a gene expression profile that is coherent and suggests a scenario related to an arrested cell cycle, senescence, and chronic inflammation, a profile that the stimulation with AM ameliorates (Liarte et al., 2020; Liarte et al., 2023). In order to see whether this AM kept in PSS at 4°C at different incubation times had a mirroring effect on gene expression profiles, we assayed the expression of critical genes involved in cell cycle regulation, inflammation, and senescence after 24-h treatment with the sets of AM in SSTC-HaCaT cells (Figure 8). First, both genes that produce cell cycle arrest, *CDKN2B* (p15) and *CDKN1A* (p21), revealed a significant sharp decrease in response to AM for all the different times tested (Figures 8A, B). In contrast, *CCNA2*, which upregulates cell cycle progression, showed significant increases with AM treatment in all cases compared to the control, explaining the AM pro-proliferative effect despite the preservation conditions (Figure 8C). Second, proinflammatory genes *IL6* and *FUCA1* exhibited significant decreases in all sets of AM kept at 4°C, suggesting a reinstatement of the SSTC-HaCaT cell inflammation to normal levels (Figures 8D, E). Last but not least, the expression of *GLB1*, a cell senescence marker, also showed a clear downregulation when challenged with AM in all preservation conditions (Figure 8F). These data suggest that preserved AM produced a long-term response (after 24 h treatment) that changed the entire expression profile to a regenerative one. In addition, the data support the notion that AM had not lost its restorative effects on the chronified status of SSTC-HaCaT cells after the freeze–thawing cycle, followed by different times of preincubation at challenging conditions.

## Discussion

In this article, we have revealed how the therapeutic effects of amniotic membrane can be preserved after a freeze–thawing cycle during different preservation times at low temperatures by using different cell models that reproduce some aspects of wounds and chronic wounds: HaCaT and SSTC-HaCaT cells.

The interest in preserving perinatal derivatives for further clinical use is not new. Perinatal derivative cryopreservation is widely used in assisted reproductive therapies, stem cell-based therapies, and tissue and organ transplantation (Chatterjee et al., 2016). Well-established freezing protocols are mandatory to respect cell survival and functionality. For instance, freezing protocols, long-term storage, and thawing are known to alter the genetic expression profile of both tissues and cells by inducing epigenetic changes such as DNA methylation or histone modification, events that can either hamper their functionality or produce therapeutic potential loss (Chatterjee et al., 2016; Downing et al., 2013; Roberts et al., 2016). In our experiments, AM is first stored in liquid nitrogen (−196°C) with the cryoprotectant DMSO and then thawed and washed three times in physiological saline solution, an established protocol that allowed us to study AM therapeutic effects in our *in vitro* models (Zelen et al., 2015; Insausti et al., 2010; Alcaraz et al., 2015; Bernabe-Garcia et al., 2017; Ruiz-Canada et al., 2018; Castellanos et al., 2017; Pipino et al., 2022; Liarte et al., 2023). Interestingly, some authors reported that freezing AM pieces at −80°C by using glycerol as a cryoprotectant does not affect AM tissue integrity and cytokine production, even when keeping AM at −80°C for months (Thomasen et al., 2009; Thomasen et al., 2011).

A clinical trial where cryopreserved AM was tested on complex non-healing wounds showed a significant effect on promoting wound closure, with 62% of the wounds studied almost healed (Svobodova et al., 2022). Other authors have developed different engineered AM strategies, such as decellularization, pulverization, or attaching AM to biocompatible scaffolds or hydrogels (Leal-Marin et al., 2021). These methods have been developed to avoid the freeze-thawing cycle; however, the efficacy of each method varies, resulting in different outcomes depending on each treatment goal. For example, it has been shown that air-dried AM is not as efficient as cryopreserved AM for corneal regeneration (Thomasen et al., 2009). However, in the case of foot ulcers, it has been shown that the treatment with lyopreserved AM, when compared with cryopreserved AM, exhibits similar therapeutic features that allow wound closure, although the wounds required more time to heal (Davis et al., 2020).

Despite the evidence on cryopreserved AM therapeutic effects, the results of this study lead us to believe that a freeze cycle for AM in cryoprotectant solutions is not mandatory because the AM could be delivered within a reasonable time, between 24 h and 48 h, to its destination at the healthcare facility. However, cryopreservation is inevitable due to the time required to perform a range of obligatory safety tests on the donor for possible human virus contamination (Pogozhykh et al., 2020). Nevertheless, once thawed, the transport of cool PSS would allow faster and easier handling and delivery without dry ice and also a faster application as AM kept in PSS can be applied directly on the wound. In addition, a sterile physiologic saline solution, which is an abundant, cheap resource in hospitals, is sufficient to carry the AM. In our tested conditions, an important number of epithelial cells in the AM were resistant to trypan blue staining, suggesting a high degree of integrity at the epithelial level that can be easily supposed for the mesenchymal cells of the AM. With this setup, we were unable to distinguish the contribution of epithelial vs. mesenchymal cells to the different tested potency assays. Future research is desirable to know the contribution of each cell type.

In our lab, different experimental settings have been developed to study the therapeutic effects of AM on different aspects of wound healing. Briefly, cell migration assays, *in situ* cell protein expression assays, gene and protein expression assays, and proliferation assays, among others, have been developed to cover a wide spectrum of biological effects contributing to the wound-healing phenomenon (Alcaraz et al., 2015; Bernabe-Garcia et al., 2017; Ruiz-Canada et al., 2018; Pipino et al., 2022; Liarte et al., 2023). The scratch wound healing assays with serum-starved HaCaT cells showed that AM activity is safeguarded even after keeping it in PSS at 4°C for 48 h, which may allow for a successful AM application after delivery to healthcare facilities. Moreover, AM activity on cell migration was preserved regardless of temperature and showed better performance than the thawed AM pieces at 37°C in serum-deprived DMEM. A plausible explanation for this outcome is that at higher temperatures (37°C), AM metabolism is accelerated, thus enhancing maintenance mechanisms inside amniotic cells such as autophagy (Li et al., 2023; Liu et al., 2024; Sato et al., 2024). In this sense, many of the cytokines and growth factors that AM releases could be processed and/or degraded, thus decreasing their bioavailability for keratinocytes. Therefore, studying the potential mechanisms behind this finding could be addressed in future publications.

Note that the presence of 4% human serum albumin in the preservation media did not exert any additional advantages on cell migration because its performance on scratch assays was worse than PSS without albumin. Regarding ERK1/2 and c-Jun blot studies, although PSS with albumin showed higher activation levels on both p-ERK1/2 and p-c-Jun, it seems that the addition of albumin is not critical because significant serum-starved HaCaT cell migration was achieved on scratch assays with AM on albumin-deprived PSS. Strikingly, there was a better performance of AM preserved in PSS at 4°C for 48 h on cell migration than standard AM (AM (C)). It is worth noting that the cell migration ability of AM preserved in PSS at 4°C was also evidenced by c-Jun staining in the HaCaT cell migration front, which revealed that AM preserved for 24 h and 48 h produced more overexpressing c-Jun cells. Thus, they increased the number of migrating cells along the wound gap. This type of behaviour has been observed in previous publications, where c-Jun displayed a similar outcome, characterized by cell recruitment and a gradient of c-Jun expression, where the highest levels appear in the cells at the wound edge when stimulated with AM or oleanolic acid (Alcaraz et al., 2015; Ruiz-Canada et al., 2018; Stelling-Ferez et al., 2022; Stelling-Férez et al., 2023). Although saline solution is very common in clinical applications, its use is widely discussed in many publications because it could trigger undesirable side effects on renal function (Blumberg et al., 2018; Awad et al., 2008). However, in our method of applying AM, PSS is used only to maintain AM pieces in optimal conditions before delivery to the final destination. It is not used later because AM pieces are intended for topical application on wounded skin (Insausti et al., 2010).

In terms of HaCaT cell proliferation, AM preserved in PSS at 4°C strengthens the phosphorylation of histone H3 in SS-HaCaT cells, which constitutes a post-translational mark that indicates mitotic entry and progression (Delcuve et al., 2009). It should be noted that when p-histone H3 quantification was studied among image sectors, this increase was strongly significant in sectors 2 and 3. This phenomenon also occurs with standard AM, with the activation of histone H3 happening deeper in the wound (Liarte et al., 2023). This outcome may be explained by the proliferation of cells deeper in the wound that allows the progression of cells to the wound edge, where the cells are recruited to migrate and take up the wound gap.

Regarding cell cycle arrest, AM kept in PSS at 4°C was able to rescue the cell cycle of both serum-starved HaCaT and SSTC-HaCaT cells, reversing the impaired phenotype. Indeed, we have disclosed in previous studies that although EGF has a strong effect on cell migration (Alcaraz et al., 2015), EGF was unable to reverse G1-cell cycle arrest induced by TGF-β1 (Liarte et al., 2020). In contrast, additionally to improving cell migration, preserved AM reduced G1 and increased S populations in both SS-HaCaT and SSTC-HaCaT cells, regardless of incubation time, temperature, TGF-β1 presence, or media.

The lack of AM activity loss at tested temperatures (20°C and 4°C) led us to believe that AM can be easily transported to healthcare centers without defined or expensive preservation media, considering temperature as a parameter to bear in mind. Nevertheless, more research is needed to assay AM activity in other cell models that are closer to the skin keratinocyte phenotype, such as N/TERT keratinocytes (Smits et al., 2017), because HaCaT cells have aneuploidy and show aberrant responses to growth factors (Rikken et al., 2020). Moreover, an *in vivo* study is still needed to finally demonstrate its therapeutic potential.

In this article, we have shown different *in vitro* assays indicating that using physiological saline solution for AM preservation is an effective measure to prolong its out-of-the-freezer life for clinical applications. Thus, the preservation protocol for long periods at 4°C not only maintains AM properties but also seems to make them more effective. Moreover, the use of PSS, a common solution for medical applications, did not negatively impact AM therapeutic properties in our *in vitro* keratinocyte model.

In conclusion, AM could be stored and delivered in PSS at 4°C from the hospital to healthcare facilities, a very easy alternative. This would be notably beneficial for the treatment of patients with wounds, allowing a better clinical practice in both small hospitals and healthcare centers, where AM treatment for wounds should be available.

## Data Availability

The original contributions presented in the study are included in the article/Supplementary Material; further inquiries can be directed to the corresponding author.

## References

[B1] Alcaraz A. Mrowiec A. Insausti C. L. Bernabe-Garcia A. Garcia-Vizcaino E. M. Lopez-Martinez M. C. (2015). Amniotic membrane modifies the genetic program induced by TGFß, stimulating keratinocyte proliferation and migration in chronic wounds. PLoS One 10 (8), e0135324. 10.1371/journal.pone.0135324 26284363 PMC4540284

[B2] Awad S. Allison S. P. Lobo D. N. (2008). The history of 0.9% saline. Clin. Nutr. 27 (2), 179–188. 10.1016/j.clnu.2008.01.008 18313809

[B3] Ball V. Younggren B. N. (2007). Emergency management of difficult wounds: part I. Emerg. Med. Clin. North Am. 25 (1), 101–121. 10.1016/j.emc.2007.01.003 17400075

[B4] Bennett B. L. Sasaki D. T. Murray B. W. O'Leary E. C. Sakata S. T. Xu W. (2001). SP600125, an anthrapyrazolone inhibitor of Jun N-terminal kinase. Proc. Natl. Acad. Sci. U. S. A. 98 (24), 13681–13686. 10.1073/pnas.251194298 11717429 PMC61101

[B5] Bernabe-Garcia A. Liarte S. Moraleda J. M. Castellanos G. Nicolas F. J. (2017). Amniotic membrane promotes focal adhesion remodeling to stimulate cell migration. Sci. Rep. 7 (1), 15262. 10.1038/s41598-017-15509-z 29127427 PMC5681678

[B6] Bihari S. Wiersema U. F. Schembri D. De Pasquale C. G. Dixon D. L. Prakash S. (1985). Bolus intravenous 0.9% saline, but not 4% albumin or 5% glucose, causes interstitial pulmonary edema in healthy subjects. J. Appl. Physiol. 119 (7), 783–792. 10.1152/japplphysiol.00356.2015 26228998

[B7] Blair M. J. Jones J. D. Woessner A. E. Quinn K. P. (2020). Skin structure-function relationships and the wound healing response to intrinsic aging. Adv. Wound Care (New Rochelle) 9 (3), 127–143. 10.1089/wound.2019.1021 31993254 PMC6985772

[B8] Blumberg N. Cholette J. M. Pietropaoli A. P. Phipps R. Spinelli S. L. Eaton M. P. (2018). 0.9% NaCl (Normal Saline) - perhaps not so normal after all? Transfus. Apher. Sci. 57 (1), 127–131. 10.1016/j.transci.2018.02.021 29523397 PMC5899644

[B9] Bonifant H. Holloway S. (2019). A review of the effects of ageing on skin integrity and wound healing. Br. J. Community Nurs. 24 (Suppl. 3), S28–S33. 10.12968/bjcn.2019.24.sup3.s28 30817191

[B10] Bos M. Mendelsohn J. Kim Y. M. Albanell J. Fry D. W. Baselga J. (1997). PD153035, a tyrosine kinase inhibitor, prevents epidermal growth factor receptor activation and inhibits growth of cancer cells in a receptor number-dependent manner. Clin. Cancer Res. 3 (11), 2099–2106. 9815602

[B11] Burgess J. L. Wyant W. A. Abdo Abujamra B. Kirsner R. S. Jozic I. (2021). Diabetic wound-healing science. Med. Kaunas. 57 (10), 1072. 10.3390/medicina57101072 34684109 PMC8539411

[B12] Calabrese E. J. Dhawan G. Kapoor R. Agathokleous E. Calabrese V. (2022). Hormesis: wound healing and keratinocytes. Pharmacol. Res. 183, 106393. 10.1016/j.phrs.2022.106393 35961478

[B13] Castellanos G. Bernabe-Garcia A. Moraleda J. M. Nicolas F. J. (2017). Amniotic membrane application for the healing of chronic wounds and ulcers. Placenta 59, 146–153. 10.1016/j.placenta.2017.04.005 28413063

[B14] Chatterjee A. Saha D. Glasmacher B. Hofmann N. (2016). Chilling without regrets: deciphering the effects of cryopreservation on the epigenetic properties of frozen cells will benefit the applications of cryo-technology. EMBO Rep. 17 (3), 292–295. 10.15252/embr.201642069 26882559 PMC4772988

[B15] Davis K. E. Killeen A. L. Farrar D. Raspovic K. M. Berriman-Rozen Z. D. Malone M. (2020). Lyopreserved amniotic membrane is cellularly and clinically similar to cryopreserved construct for treating foot ulcers. Int. Wound J. 17 (6), 1893–1901. 10.1111/iwj.13479 32820605 PMC7754413

[B16] Delcuve G. P. Rastegar M. Davie J. R. (2009). Epigenetic control. J. Cell Physiol. 219 (2), 243–250. 10.1002/jcp.21678 19127539

[B17] Diaz-Herrera M. A. Martinez-Riera J. R. Verdu-Soriano J. Capillas-Perez R. M. Pont-Garcia C. Tenllado-Perez S. (2021). Multicentre study of chronic wounds point prevalence in primary Health care in the southern metropolitan area of Barcelona. J. Clin. Med. 10 (4), 797. 10.3390/jcm10040797 33669397 PMC7920417

[B18] Downing T. L. Soto J. Morez C. Houssin T. Fritz A. Yuan F. (2013). Biophysical regulation of epigenetic state and cell reprogramming. Nat. Mater 12 (12), 1154–1162. 10.1038/nmat3777 24141451 PMC9675045

[B19] El-Amawy H. S. Sarsik S. M. (2021). Saline in Dermatology: a literature review. J. Cosmet. Dermatol 20 (7), 2040–2051. 10.1111/jocd.13813 33098717

[B20] Eming S. A. Krieg T. Davidson J. M. (2007). Inflammation in wound repair: molecular and cellular mechanisms. J. Invest Dermatol 127 (3), 514–525. 10.1038/sj.jid.5700701 17299434

[B21] Eming S. A. Martin P. Tomic-Canic M. (2014). Wound repair and regeneration: mechanisms, signaling, and translation. Sci. Transl. Med. 6 (265), 265sr6. 10.1126/scitranslmed.3009337 25473038 PMC4973620

[B22] Ernst O. Zor T. (2010). Linearization of the bradford protein assay. J. Vis. Exp. (38), 1918. 10.3791/1918 20386536 PMC3164080

[B23] Ferreira M. C. Tuma P. Jr. Carvalho V. F. Kamamoto F. (2006). Complex wounds. Clin. (Sao Paulo) 61 (6), 571–578. 10.1590/s1807-59322006000600014 17187095

[B24] Flores A. I. Pipino C. Jerman U. D. Liarte S. Gindraux F. Kreft M. E. (2022). Perinatal derivatives: how to best characterize their multimodal functions *in vitro*. Part C: inflammation, angiogenesis, and wound healing. Front. Bioeng. Biotechnol. 10, 965006. 10.3389/fbioe.2022.965006 35992360 PMC9386263

[B25] Gindraux F. Hofmann N. Agudo-Barriuso M. Antica M. Couto P. S. Dubus M. (2022). Perinatal derivatives application: identifying possibilities for clinical use. Front. Bioeng. Biotechnol. 10, 977590. 10.3389/fbioe.2022.977590 36304904 PMC9595339

[B26] Gramignoli R. Hofmann N. Agudo-Barriuso M. Antica M. Flores A. I. Girandon L. (2024). Expert revision of key elements for clinical-grade production and qualification of perinatal derivatives. Stem Cells Transl. Med. 13 (1), 14–29. 10.1093/stcltm/szad068 38071447 PMC10785218

[B27] Greenhalgh D. G. (2003). Wound healing and diabetes mellitus. Clin. Plast. Surg. 30 (1), 37–45. 10.1016/s0094-1298(02)00066-4 12636214

[B28] Herdegen T. Skene P. Bahr M. (1997). The c-Jun transcription factor--bipotential mediator of neuronal death, survival and regeneration. Trends Neurosci. 20 (5), 227–231. 10.1016/s0166-2236(96)01000-4 9141200

[B29] Hofmann N. Lafarge X. Antica M. Ferry N. Girandon L. Gramignoli R. (2023). Expert consideration on regulatory aspects for perinatal derivatives in clinical settings. Stem Cells Transl. Med. 12 (5), 258–265. 10.1093/stcltm/szad017 37027834 PMC10184691

[B30] Insausti C. L. Alcaraz A. Garcia-Vizcaino E. M. Mrowiec A. Lopez-Martinez M. C. Blanquer M. (2010). Amniotic membrane induces epithelialization in massive posttraumatic wounds. Wound Repair Regen. 18 (4), 368–377. 10.1111/j.1524-475x.2010.00604.x 20636551

[B31] Janis J. E. Harrison B. (2016). Wound healing: Part I. Basic science. Basic Sci. Plast Reconstr Surg 138 (3 Suppl. l), 9S–17S. 10.1097/prs.0000000000002773 27556781

[B32] Jie J. Yang J. He H. Zheng J. Wang W. Zhang L. (2018). Tissue remodeling after ocular surface reconstruction with denuded amniotic membrane. Sci. Rep. 8 (1), 6400. 10.1038/s41598-018-24694-4 29686390 PMC5913251

[B33] Kasuya A. Tokura Y. (2014). Attempts to accelerate wound healing. J. Dermatol Sci. 76 (3), 169–172. 10.1016/j.jdermsci.2014.11.001 25468357

[B34] Koh J. W. Shin Y. J. Oh J. Y. Kim M. K. Ko J. H. Hwang J. M. (2007). The expression of TIMPs in cryo-preserved and freeze-dried amniotic membrane. Curr. Eye Res. 32 (7-8), 611–616. 10.1080/02713680701459441 17852184

[B35] Leal-Marin S. Kern T. Hofmann N. Pogozhykh O. Framme C. Borgel M. (2021). Human Amniotic Membrane: a review on tissue engineering, application, and storage. J. Biomed. Mater Res. B Appl. Biomater. 109 (8), 1198–1215. 10.1002/jbm.b.34782 33319484

[B36] Li F. X. Liu J. J. Xu F. Shan S. K. Zheng M. H. Lei L. M. (2023). Cold exposure protects against medial arterial calcification development via autophagy. J. Nanobiotechnology 21 (1), 226. 10.1186/s12951-023-01985-1 37461031 PMC10351118

[B37] Li G. Gustafson-Brown C. Hanks S. K. Nason K. Arbeit J. M. Pogliano K. (2003). c-Jun is essential for organization of the epidermal leading edge. Dev. Cell 4 (6), 865–877. 10.1016/s1534-5807(03)00159-x 12791271

[B38] Liarte S. Bernabe-Garcia A. Armero-Barranco D. Nicolas F. J. (2018). Microscopy based methods for the assessment of epithelial cell migration during *in vitro* wound healing. J. Vis. Exp. 131, 56799. 10.3791/56799 29364245 PMC5908412

[B39] Liarte S. Bernabe-Garcia A. Nicolas F. J. (2020). Human skin keratinocytes on sustained TGF-β stimulation reveal partial EMT features and weaken growth arrest responses. Cells 9 (1), 255. 10.3390/cells9010255 31968599 PMC7017124

[B40] Liarte S. Bernabe-Garcia A. Rodriguez-Valiente M. Moraleda J. M. Castellanos G. Nicolas F. J. (2023). Amniotic membrane restores chronic wound features to normal in a keratinocyte TGF-β-chronified cell model. Int. J. Mol. Sci. 24 (7), 6210. 10.3390/ijms24076210 37047181 PMC10094701

[B41] Liu B. Liu L. Liu Y. (2024). Targeting cell death mechanisms: the potential of autophagy and ferroptosis in hepatocellular carcinoma therapy. Front. Immunol. 15, 1450487. 10.3389/fimmu.2024.1450487 39315094 PMC11416969

[B42] Neuhaus K. Meuli M. Koenigs I. Schiestl C. (2013). Management of “difficult” wounds. Eur. J. Pediatr. Surg. 23 (5), 365–374. 10.1055/s-0033-1354588 24008551

[B43] Nguyen A. V. Soulika A. M. (2019). The dynamics of the skin's immune system. Int. J. Mol. Sci. 20 (8), 1811. 10.3390/ijms20081811 31013709 PMC6515324

[B44] Okonkwo U. A. DiPietro L. A. (2017). Diabetes and wound angiogenesis. Int. J. Mol. Sci. 18 (7), 1419. 10.3390/ijms18071419 28671607 PMC5535911

[B45] Paluch B. E. Naqash A. R. Brumberger Z. Nemeth M. J. Griffiths E. A. (2016). Epigenetics: a primer for clinicians. Blood Rev. 30 (4), 285–295. 10.1016/j.blre.2016.02.002 26969414 PMC5737767

[B46] Patel S. Srivastava S. Singh M. R. Singh D. (2019). Mechanistic insight into diabetic wounds: pathogenesis, molecular targets and treatment strategies to pace wound healing. Biomed. Pharmacother. 112, 108615. 10.1016/j.biopha.2019.108615 30784919

[B47] Pfeffer F. von Dobschuetz E. Riediger H. Moosmann C. Hopt U. T. (2004). The non-healing wound. MMW Fortschr Med. 146 (44), 45–48. 15566249

[B48] Pipino C. Bernabe-Garcia A. Cappellacci I. Stelling-Ferez J. Di Tomo P. Santalucia M. (2022). Effect of the human amniotic membrane on the umbilical vein endothelial cells of gestational diabetic mothers: new insight on inflammation and angiogenesis. Front. Bioeng. Biotechnol. 10, 854845. 10.3389/fbioe.2022.854845 35866032 PMC9294233

[B49] Pogozhykh O. Hofmann N. Gryshkov O. von Kaisenberg C. Mueller M. Glasmacher B. (2020). Repeated freezing procedures preserve structural and functional properties of amniotic membrane for application in ophthalmology. Int. J. Mol. Sci. 21 (11), 4029. 10.3390/ijms21114029 32512889 PMC7312941

[B50] Reinke J. M. Sorg H. (2012). Wound repair and regeneration. Eur. Surg. Res. 49 (1), 35–43. 10.1159/000339613 22797712

[B51] Ridiandries A. Tan J. T. M. Bursill C. A. (2018). The role of chemokines in wound healing. Int. J. Mol. Sci. 19 (10), 3217. 10.3390/ijms19103217 30340330 PMC6214117

[B52] Rikken G. Niehues H. van den Bogaard E. H. (2020). Organotypic 3D skin models: human epidermal equivalent cultures from primary keratinocytes and immortalized keratinocyte cell lines. Methods Mol. Biol. 2154, 45–61. 10.1007/978-1-0716-0648-3_5 32314207

[B53] Roberts S. A. Hann M. Brison D. R. (2016). Factors affecting embryo viability and uterine receptivity: insights from an analysis of the UK registry data. Reprod. Biomed. Online 32 (2), 197–206. 10.1016/j.rbmo.2015.11.002 26655652

[B54] Rodrigues M. Kosaric N. Bonham C. A. Gurtner G. C. (2019). Wound healing: a cellular perspective. Physiol. Rev. 99 (1), 665–706. 10.1152/physrev.00067.2017 30475656 PMC6442927

[B55] Ruiz-Canada C. Bernabe-Garcia A. Liarte S. Insausti C. L. Angosto D. Moraleda J. M. (2018). Amniotic membrane stimulates cell migration by modulating transforming growth factor‐β signalling. J. Tissue Eng. Regen. Med. 12 (3), 808–820. 10.1002/term.2501 28621502

[B56] Ruiz-Canada C. Bernabe-Garcia A. Liarte S. Rodriguez-Valiente M. Nicolas F. J. (2021). Chronic wound healing by amniotic membrane: TGF-β and EGF signaling modulation in Re-epithelialization. Front. Bioeng. Biotechnol. 9, 689328. 10.3389/fbioe.2021.689328 34295882 PMC8290337

[B57] Sato A. Inayoshi S. Kitawaki K. Mihara R. Yoneda K. Ito-Inaba Y. (2024). Autophagy is suppressed by low temperatures and is dispensable for cold acclimation in Arabidopsis. Physiol. Plant 176 (4), e14409. 10.1111/ppl.14409 38973450

[B58] Shang J. Lu S. Jiang Y. Zhang J. (2016). Allosteric modulators of MEK1: drug design and discovery. Chem. Biol. Drug Des. 88 (4), 485–497. 10.1111/cbdd.12780 27115708

[B59] Singer A. J. Clark R. A. (1999). Cutaneous wound healing. N. Engl. J. Med. 341 (10), 738–746. 10.1056/nejm199909023411006 10471461

[B60] Smits J. P. H. Niehues H. Rikken G. van Vlijmen-Willems I. van de Zande G. Zeeuwen P. (2017). Immortalized N/TERT keratinocytes as an alternative cell source in 3D human epidermal models. Sci. Rep. 7 (1), 11838. 10.1038/s41598-017-12041-y 28928444 PMC5605545

[B61] Stelling-Ferez J. Cappellacci I. Pandolfi A. Gabaldon J. A. Pipino C. Nicolas F. J. (2023). Oleanolic acid rescues critical features of umbilical vein endothelial cells permanently affected by hyperglycemia. Front. Endocrinol. (Lausanne) 14, 1308606. 10.3389/fendo.2023.1308606 38192424 PMC10773851

[B62] Stelling-Ferez J. Gabaldon J. A. Nicolas F. J. (2022). Oleanolic acid stimulation of cell migration involves a biphasic signaling mechanism. Sci. Rep. 12 (1), 15065. 10.1038/s41598-022-17553-w 36064555 PMC9445025

[B63] Stelling-Férez J. López-Miranda S. Gabaldón J. A. Nicolás F. J. (2023). Oleanolic acid complexation with cyclodextrins improves its cell bio-availability and biological activities for cell migration. Int. J. Mol. Sci. 24 (19), 14860. 10.3390/ijms241914860 37834307 PMC10573973

[B64] Svobodova A. Horvath V. Smeringaiova I. Cabral J. V. Zemlickova M. Fiala R. (2022). The healing dynamics of non-healing wounds using cryo-preserved amniotic membrane. Int. Wound J. 19 (5), 1243–1252. 10.1111/iwj.13719 34791774 PMC9284646

[B65] Thomasen H. Pauklin M. Noelle B. Geerling G. Vetter J. Steven P. (2011). The effect of long-term storage on the biological and histological properties of cryopreserved amniotic membrane. Curr. Eye Res. 36 (3), 247–255. 10.3109/02713683.2010.542267 21275517

[B66] Thomasen H. Pauklin M. Steuhl K. P. Meller D. (2009). Comparison of cryopreserved and air-dried human amniotic membrane for ophthalmologic applications. Graefes Arch. Clin. Exp. Ophthalmol. 247 (12), 1691–1700. 10.1007/s00417-009-1162-y 19693529

[B67] Toda A. Okabe M. Yoshida T. Nikaido T. (2007). The potential of amniotic membrane/amnion-derived cells for regeneration of various tissues. J. Pharmacol. Sci. 105 (3), 215–228. 10.1254/jphs.cr0070034 17986813

[B68] Wang P. H. Huang B. S. Horng H. C. Yeh C. C. Chen Y. J. (2018). Wound healing. J. Chin. Med. Assoc. 81 (2), 94–101. 10.1016/j.jcma.2017.11.002 29169897

[B69] Xu X. Liu Y. Cui Z. F. (2014). Effects of cryopreservation on human mesenchymal stem cells attached to different substrates. J. Tissue Eng. Regen. Med. 8 (8), 664–672. 10.1002/term.1570 25066447

[B70] Yuceer R. O. Baspinar S. (2024). Investigation of Ki67 and phospho-histone H3 expressions in urothelial carcinoma of the bladder by immunohistochemical method. Cureus 16 (2), e55297. 10.7759/cureus.55297 38558732 PMC10981782

[B71] Yue C. Guo Z. Luo Y. Yuan J. Wan X. Mo Z. (2020). c-Jun overexpression accelerates wound healing in diabetic rats by human umbilical cord-derived mesenchymal stem cells. Stem Cells Int. 2020, 1–10. 10.1155/2020/7430968 32399050 PMC7201444

[B72] Zelen C. M. Snyder R. J. Serena T. E. Li W. W. (2015). The use of human amnion/chorion membrane in the clinical setting for lower extremity repair: a review. Clin. Podiatr. Med. Surg. 32 (1), 135–146. 10.1016/j.cpm.2014.09.002 25440424

