# Supplementary figures and images for: Refrigerated amniotic membrane maintains its therapeutic qualities for 48 hours
Source: Front Bioeng Biotechnol. 2024 Nov 6;12:1455397. doi: 10.3389/fbioe.2024.1455397 (PMC11576280; doi:10.3389/fbioe.2024.1455397)

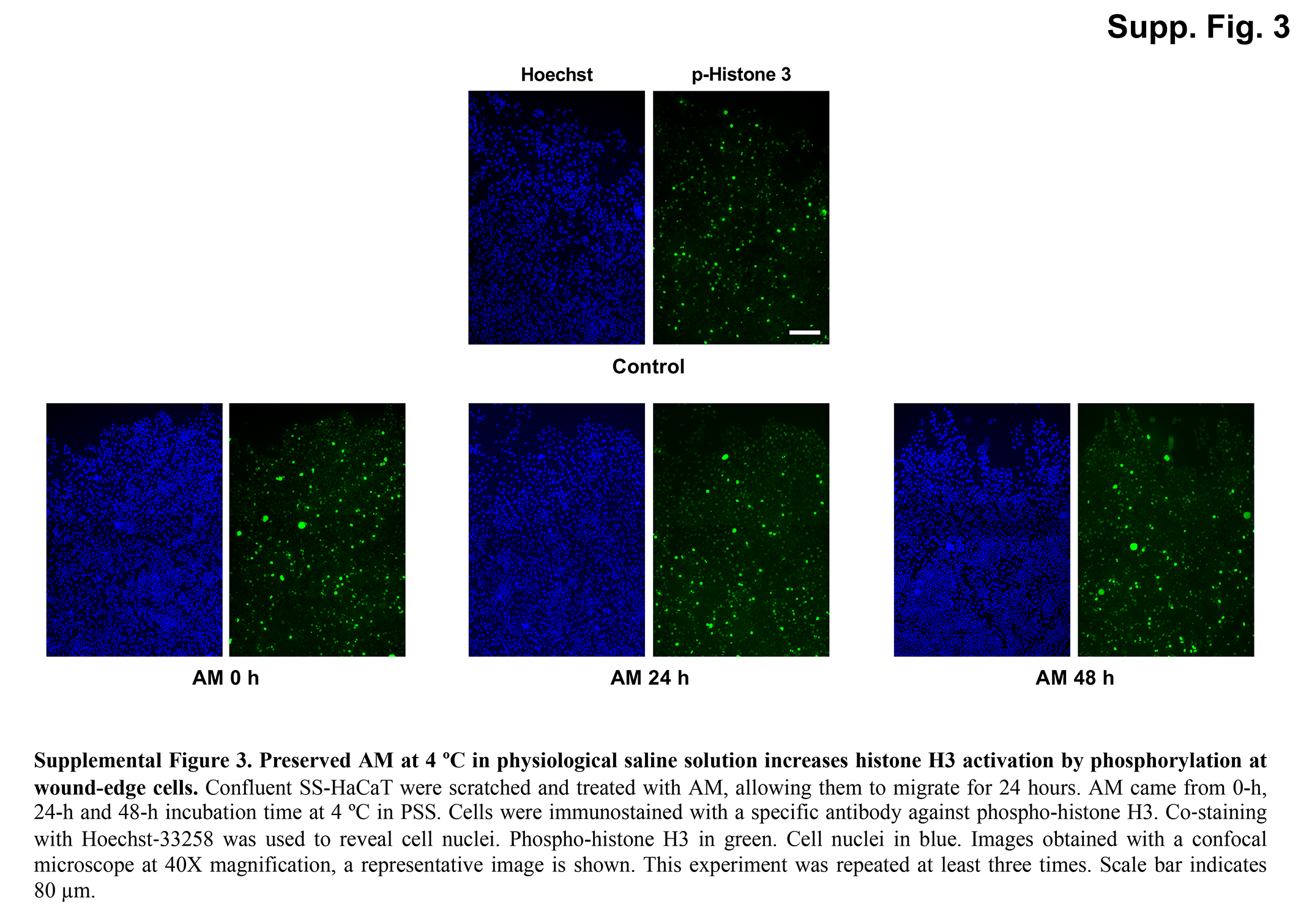

Supplement: Supplementary file 1 [file Image3.TIF]

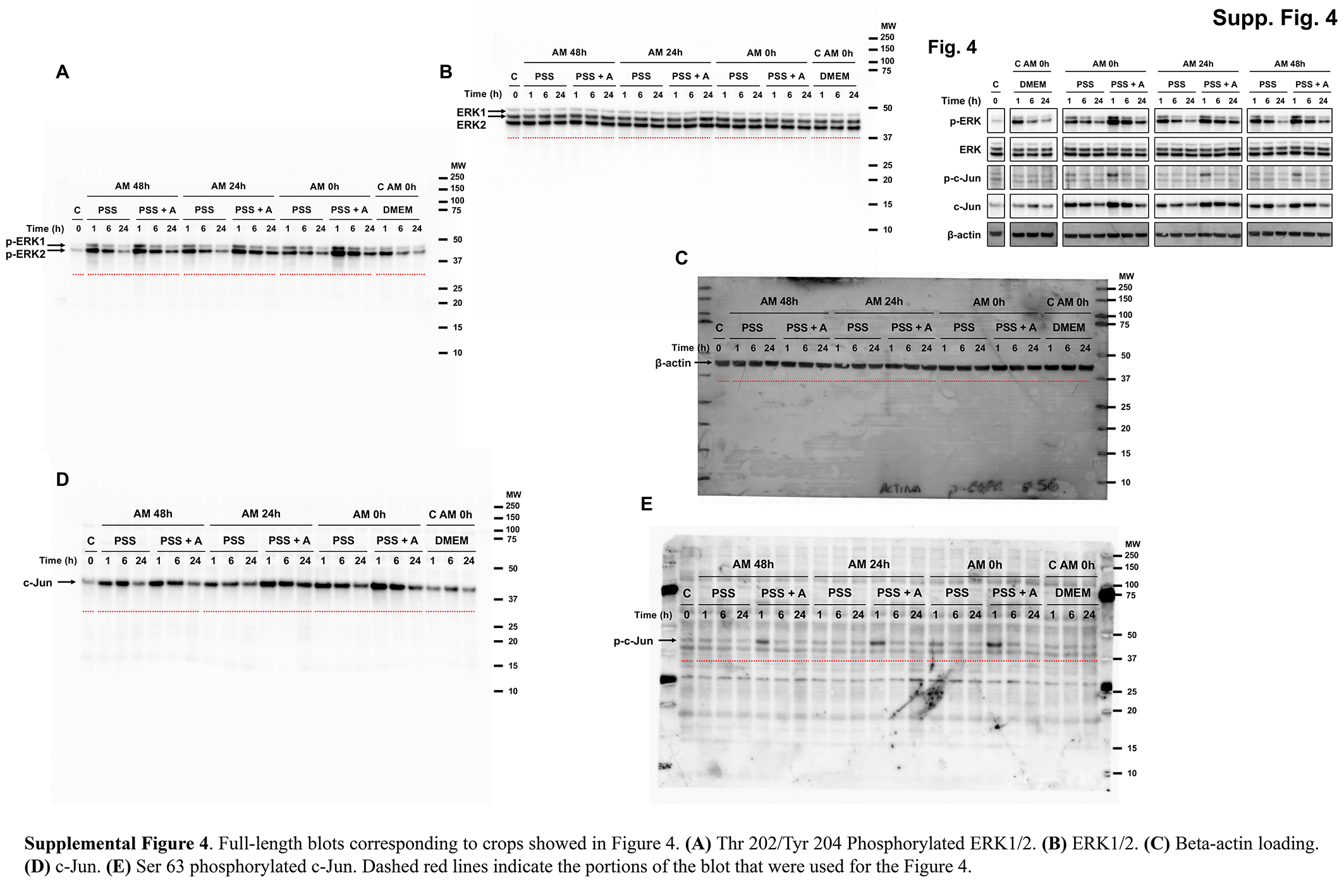

Supplement: Supplementary file 2 [file Image4.TIF]

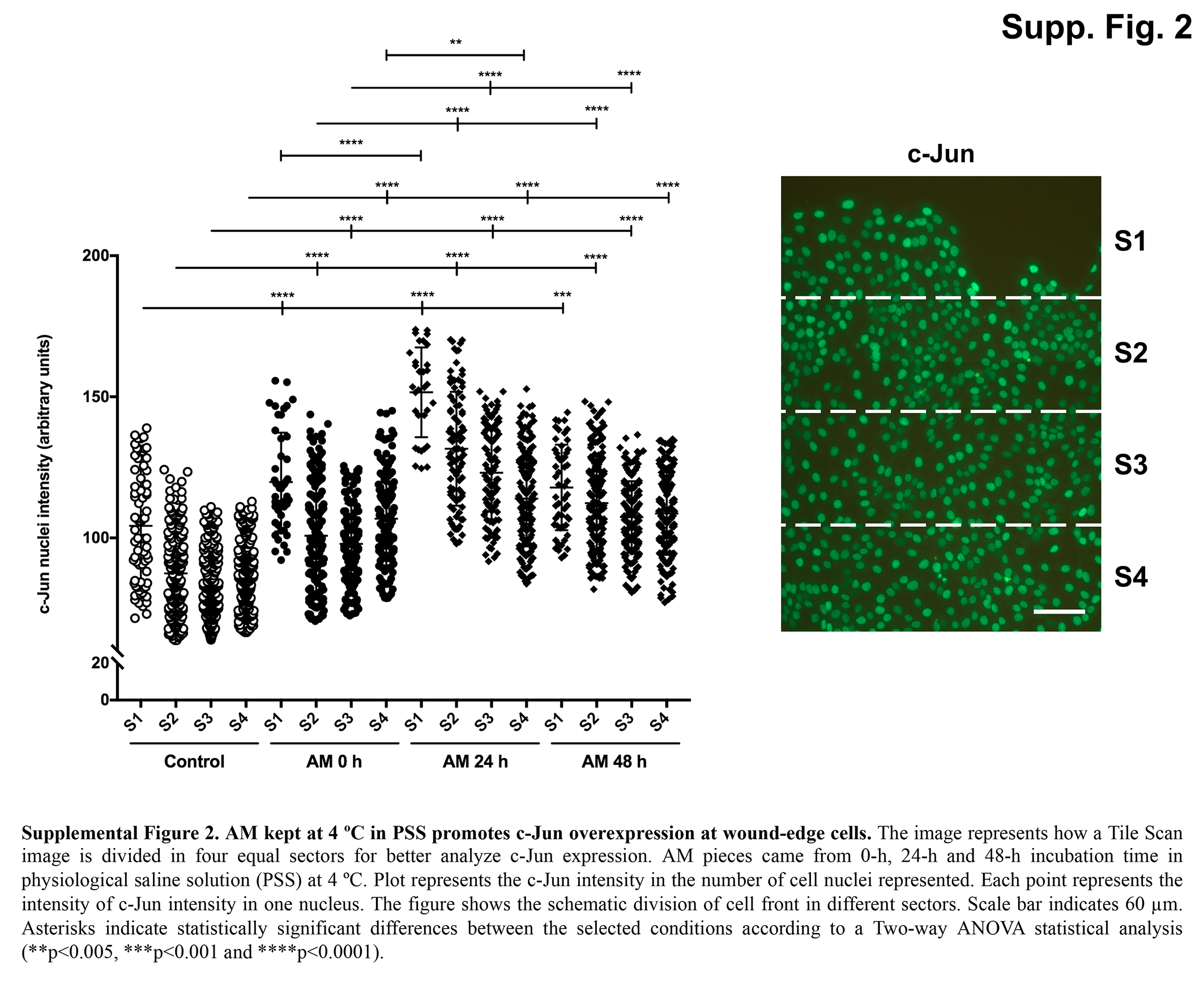

Supplement: Supplementary file 3 [file Image2.TIF]

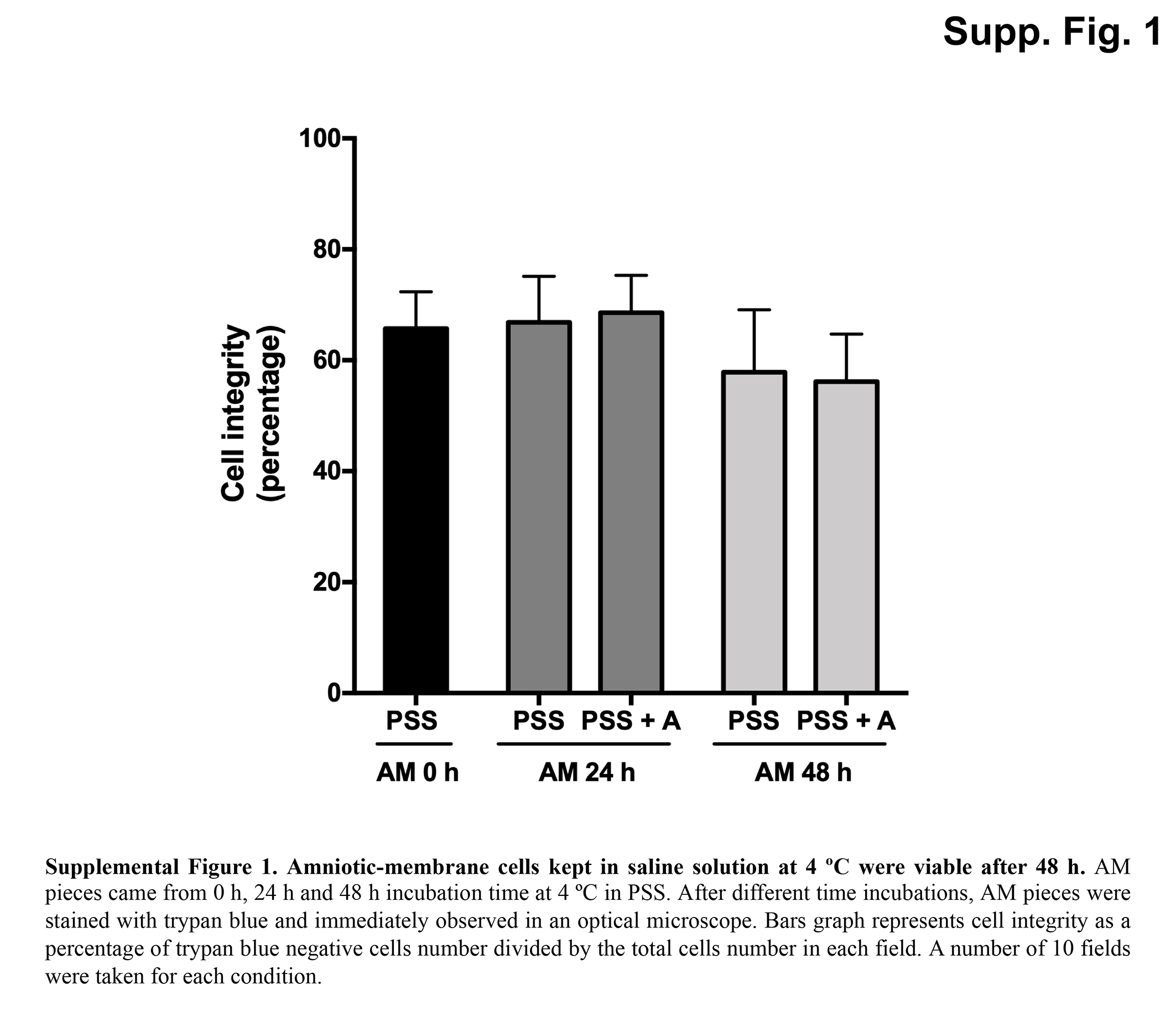

Supplement: Supplementary file 4 [file Image1.TIF]
